# Human fertility in relation to education, economy, religion, contraception, and family planning programs

**DOI:** 10.1186/s12889-020-8331-7

**Published:** 2020-02-22

**Authors:** Frank Götmark, Malte Andersson

**Affiliations:** Department of Biological and Environmental Sciences, University of Gothenburg, Göteborg, Sweden

**Keywords:** Human population, Population policy, Demography, Sustainability, Economy, Education, Family planning, Schooling, Religiosity

## Abstract

**Background:**

The world population is expected to increase greatly this century, aggravating current problems related to climate, health, food security, biodiversity, energy and other vital resources. Population growth depends strongly on total fertility rate (TFR), but the relative importance of factors that influence fertility needs more study.

**Methods:**

We analyze recent levels of fertility in relation to five factors: education (mean school years for females), economy (Gross Domestic Product, GDP, per capita), religiosity, contraceptive prevalence rate (CPR), and strength of family planning programs. We compare six global regions: E Europe, W Europe and related countries, Latin America and the Caribbean, the Arab States, Sub-Saharan Africa, and Asia. In total, 141 countries are included in the analysis. We estimate the strength of relationships between TFR and the five factors by correlation or regression and present the results graphically.

**Results:**

In decreasing order of strength, fertility (TFR) correlates negatively with education, CPR, and GDP per capita, and positively with religiosity. Europe deviates from other regions in several ways, e.g. TFR increases with education and decreases with religiosity in W Europe. TFR decreases with increasing strength of family planning programs in three regions, but only weakly so in a fourth, Sub-Saharan Africa (the two European regions lacked such programs). Most factors correlated with TFR are also correlated with each other. In particular, education correlates positively with GDP per capita but negatively with religiosity, which is also negatively related to contraception and GDP per capita.

**Conclusions:**

These results help identify factors of likely importance for TFR in global regions and countries. More work is needed to establish causality and relative importance of the factors. Our novel quantitative analysis of TFR suggests that religiosity may counteract the ongoing decline of fertility in some regions and countries.

## Background

United Nations (UN) projects that the global human population may increase from 7.8 billion in 2020 to 10.9 billion by 2100 (‘medium variant’ [1];). A 40% population increase would have strong effects on economies, food production, environment and global climate [2–5]. Understanding the causes of this extraordinary population growth is critical for many aspects of international and national planning for the future (e.g., [6]).

The total fertility rate (TFR) is a major determinant of population growth rate [1]. TFR is the average number of children women would bear, if they survive to the end of reproductive life and have the same probability of child-bearing in each age interval as currently prevails across the population. Based on observations of past and ongoing global decline in TFR, UN [1] assumes in its medium projection model that TFR in all countries will converge to near replacement level (2.1) during the decades up to 2100. However, such continued decrease in TFR should not be taken for granted [7, 8], and altered assumptions markedly change the population projections [1]. Population policies depend strongly on our limited knowledge and assumptions about how TFR is related to other factors.

The steady decline in global fertility that began about 1965 stalled in many countries from the mid-1990s ([9]; for Africa, see [10]). Limitations in contraceptive use, family planning programs [11, 12] and education [13] may be involved in the stall, impeding efforts to reduce population growth. Here we study TFR in six global regions and analyze its relation to five debated factors that are known or assumed to influence fertility: education of girls and women, economy (GDP per capita), religiosity, contraceptive use, and family planning programs. Other factors can also influence TFR [9, 14] and may be correlated with the factors analyzed here (see Discussion).

### Education

Lutz [15] suggested a rationale for population policies based on the relation of TFR to education and health. Increased education (school years) of girls and women is associated with declining fertility in many countries. Education can change family relations and childbearing decisions. More and longer education can bring about empowerment of women, later marriage, later onset of childbearing, and smaller family size (e.g. [16–19]). There is variation among countries, and the empirical record does “not support the idea that such a simple causal process operates everywhere” [20]. Nevertheless, fertility differs between more and less educated women in nearly all countries, but the precise mechanism that leads to lower TFR with longer education is not well known [21].

### Economy

Reduced family size as nations and economies develop might be due to increasing income per capita, and to trade-off between quantity and quality of children [14, 22] (review in [23]). Technology favors investment in longer education (human capital), implying higher costs of children, and opportunity costs for child-rearing women in job markets (“motherhood wage penalty”). Families are therefore expected to invest in more highly educated but fewer children, and TFR declines.

Based on theoretical models and data from European and other countries, Galor [24] analyzed four suggested causes of the demographic transition and declining TFR 1851–1915: rising income, reduced child mortality, children as old-age security, and rising demand for education. He rejected the first three (but see [25]), emphasizing the role of increasing education for fertility decline. Growing economy, industrial production and technology favored higher child quality, and hence smaller families [24]. In two studies based on countries as units, TFR was more strongly related to education than to GDP per capita [19, 26]. TFR had little relation to the level and change of GDP per capita 1960–2010, but GDP changes tended to be increasingly positive for countries at lower TFR level [27]. Lower TFR may therefore favor economic development, rather than the other way around [28, 29].

### Religion

Faith and religious authority can influence TFR at individual and country levels. For instance, at the UN population conference in Cairo 1994, Vatican and Muslim leaders opposed aspects of family planning, especially abortion and women’s autonomy [30, 31]. Increased faith has accompanied population growth in parts of the world [32, 33]. Based on the World Values Survey, Norris & Inglehart [34] ranked 73 countries as “most secular”, “moderate”, or “most religious”. Mean TFR 1970–1975 for the most secular countries was 2.8 children, for moderate 3.3 and for most religious 5.4. The corresponding values 2000–2005 were 1.8, 1.7 and 2.8. Several other studies also suggest that religiosity favors high TFR [35–37].

### Contraceptives and family planning programs

The family planning (FP) movement and FP programs emphasize women’s rights and empowerment, and the imbalance between human numbers and vital resources [38, 39]. FP programs spread information, counsel couples and make contraceptives easily available, all of which may reduce TFR. Use of modern contraceptives is important [11, 21, 40], and there is experimental evidence that FP programs increase contraceptive use and reduce TFR [41–43]. Other factors, such as education and religiosity, also influence contraceptive use (e.g. [44, 45]). After the UN Cairo conference in 1994 a concept less clearly linked to family size (“sexual and reproductive health and rights”) spread, and support for family planning declined [27, 31, 38, 46].

For our analyses, data on contraceptives (and education, economy, religion) were available from six regions, while data on FP programs were available from four regions. FP programs, potentially important in four high fertility regions, are analyzed separately from other factors, but all factors are treated in the Discussion.

## Methods

### Analytical approach

We analyse TFR at regional and country levels. Most studies analyze single factors and groups of countries [11, 14, 21, 47]. Studies that include both developing and developed countries usually deal with one or two factors (but see [19]). To our knowledge, TFR and its relations to education, economy, religion, contraception and FP programs have not been analyzed together in the major global regions, our aim here.

Many studies use countries as sample units in statistical analyses and tests, but countries may not be statistically independent units. Neighboring countries can be similar culturally, economically or politically, and also distant countries can have political and economic ties [48] and similarities in health status and social norms (e.g. [49]). Some countries may therefore form clusters of similar units, differing markedly from other clusters. Moreover, the number of countries in an area is a partly arbitrary consequence of political events, which may divide a nation into two or more (e.g. former Jugoslavia and Sudan). Countries therefore deviate from requirements of independent sample units in many probabilistic statistical methods. Using countries as units in statistical tests may therefore lead to pseudo-replication, inflated sample size and misleading results as regards probability levels [50, 51], a problem that deserves further attention from statisticians.

We therefore avoid statistical testing and multivariate statistical modeling, instead using simple correlation, regression and graphical analysis (e.g. [52]) for generating hypotheses and identifying factors of likely importance for causal analyses of TFR (see also [21, 53]). We do not analyze all countries together but group them into six global regions, forming sets of geographically or otherwise related countries that share similarities, as explained below. Among the regions we examine the extent to which TFR is related to the five factors and how the factors correlate with each other, exploring potential differences between regions.

Regions may differ systematically in unmeasured factors that affect TFR. Compared to analyzing all countries together, analyzing regions separately can then reduce the influence of unmeasured variation in the analysis, increasing the possibility of clarifying differences between the five factors studied as regards their importance for TFR. We complement this approach by analyzing differences and similarities within regions, with countries as units. We use estimates of TFR and the factors from 2005 to 2015 (see below). The results therefore concern the recent situation and help identify factors of likely importance for future causal analyses of TFR.

### Regions and countries

We included countries with available data for education, economy, religion and contraception (for FP programs, see below). Russia, China and several other countries could not be included due to lack of data. Based on 141 countries (Table 1) we established regions, taking into account geography and culture, as UNESCO [55] did in categorizing five global regions (Africa, Arab states, Asia and the Pacific, Europe and North America, Latin America and the Caribbean). We also considered shared history and degree of economic and political ties, and differ from UNESCO [55] mainly in using Eastern Europe as a separate region (motivated by spatial proximity and common history of Soviet influence). The six regions are as follows (see also Table 1):

- *Western Europe and related states (“W Europe” below)*. Countries west of the former Soviet Union, and six states with ties to W Europe historically and politically: Israel, Iceland, USA, Canada, Australia and New Zealand. In total 25 countries.
- *Eastern Europe (“E Europe” below)*. Countries in E Europe formerly linked to the Soviet Union, and also Albania, Turkey and Georgia. 20 countries.
- *Latin America and the Caribbean (“Latin America” below).* All American countries S of the US, including four in the Caribbean: Trinidad & Tobago, Jamaica, Dominican Republic and Haiti. 23 countries.
- *Arab States*. Countries in NW, N and NE Africa and in Western Asia, including Iraq and countries in the Arab peninsula but not further east.18 countries.
- *Sub-Saharan Africa*. Countries south of the Sahara (Comoros, Sudan and Mauritania are included in Arab States). 31 countries.
- *Asia*. Countries in central, E and S Asia. This is the most diverse region, with countries that to some extent share cultures and political systems, although these vary markedly. We did not divide Asia into smaller regions as they would contain too few countries for meaningful analyses. 24 countries (small Pacific island nations are excluded).

**Table 1:** Six Regions and 141 Countries used in the Analyses, with Fertility Rates (TFR) for each Country 2010–2015 (in ascending order of TFR)

Region	TFR
Western Europe and related countries	
Portugal	1,28
Spain	1,33
Greece	1,34
Cyprus	1,38
Malta	1,41
Germany	1,43
Italy	1,43
Austria	1,45
Switzerland	1,53
Luxembourg	1,55
Canada	1,61
Denmark	1,73
Netherlands	1,73
Finland	1,77
Belgium	1,78
Norway	1,82
USA	1,88
UK	1,88
Australia	1,89
Sweden	1,90
France	1,98
Iceland	1,98
Ireland	2,00
New Zeeland	2,04
Israel	3,04
Eastern Europe	
Bosnia-Herzegovina	1,31
Hungary	1,33
Poland	1,33
Slovakia	1,39
Czech Republic	1,48
Rumania	1,48
Croatia	1,49
Ukraine	1,49
Latvia	1,50
Bulgaria	1,51
Slovenia	1,58
Estonia	1,59
Serbia	1,59
Lithuania	1,59
Belarus	1,64
Armenia	1,65
Montenegro	1,71
Albania	1,71
Georgia	2,00
Turkey	2,12
Latin America and the Caribbean	
Brazil	1,78
Trinidad & Tobago	1,80
Chile	1,82
Costa Rica	1,85
Columbia	1,93
Uruguay	2,04
Jamaica	2,08
El Salvador	2,17
Mexico	2,29
Nicaragua	2,32
Argentina	2,35
Venezuela	2,40
Peru	2,50
Dominican Republic	2,53
Ecuador	2,59
Paraguay	2,60
Guyana	2,60
Panama	2,60
Belize	2,64
Honduras	2,65
Bolivia	3,04
Haiti	3,13
Guatemala	3,19
Arab States	
Lebanon	1,72
United Arab Emirates	1,82
Qatar	2,00
Kuwait	2,05
Bahrain	2,12
Tunisia	2,25
Libya	2,40
Morocco	2,60
Saudi Arabia	2,73
Algeria	2,96
Egypt	3,38
Jordan	3,60
Palestinian Terr.	4,25
Yemen	4,40
Iraq	4,55
Comoros	4,60
Sudan	4,75
Mauretania	4,88
Sub-Saharan Africa	
South Africa	2,55
Botswana	2,88
Swaziland	3,30
Namibia	3,60
Zimbabwe	4,00
Kenya	4,10
Ghana	4,18
Rwanda	4,20
Madagascar	4,40
Togo	4,69
Liberia	4,83
Sierra Leone	4,84
Congo Brazzaville	4,86
Malawi	4,88
Cameroon	4,95
Senegal	5,00
Central African Republic	5,10
Guinea	5,13
Ivory Coast	5,14
Zambia	5,20
Benin	5,22
Tanzania	5,24
Mozambique	5,45
Burkina Faso	5,65
Nigeria	5,74
Uganda	5,91
Burundi	6,00
Chad	6,31
Mali	6,35
Congo Kinshasa	6,40
Niger	7,40
Asia	
Hong Kong (China)	1,20
Singapore	1,23
South Korea	1,23
Japan	1,41
Thailand	1,53
Iran	1,75
Vietnam	1,96
Azerbaijan	2,10
Malaysia	2,11
Bangladesh	2,22
Myanmar	2,30
Nepal	2,32
Uzbekistan	2,38
India	2,44
Indonesia	2,45
Cambodia	2,70
Kazakstan	2,70
Mongolia	2,83
Laos	2,93
Philippines	3,05
Kyrgyzstan	3,12
Tajikistan	3,50
Pakistan	3,72
Afghanistan	5,26

Source: TFR based on [54]

### Data

Table 1 lists countries and TFR for the five-year period June 2010–June 2015 [54]. Data for female school years are from UNDP [56] and represent “average number of years of education received by people ages 25 and older, converted from education attainment levels using official durations of each level”. The data are means for 2011–2015. We used data for females, as theory and population policies focus on female education.

Data for GDP (Gross Domestic Product) per capita are World Bank [57] mean values for 2011–2015, in PPP (Purchasing Power Parity, International dollars, constant 2011 values). Data for religion come from standardized surveys of religiosity by Gallup, Inc. For each country a sample of 1000 respondents is drawn, and weights are assigned so the data reflect the population in terms of gender, age, education, household size, and socioeconomic status. The survey, starting in 2005, has been repeated several times in each country. We use the Gallup question “Is religion an important part of your daily life?” and the proportion of respondents in each country saying yes to this question (“yes” or “no” were the options). We use a compilation of pooled Gallup data for individual countries collected from 2005 to about 2012 ([33]: Table 1–6). The average number of respondents per country was 7567 ([33], p. 12).

As a fourth factor in our analyses of the six regions we used contraceptive prevalence rate (CPR). No data were available for family planning (FP) programs in the two European regions. CPR should bear some relation to FP programs but may not reflect their strength, which we analyze separately (see below). In a global report [58], CPR is recorded for “generally married or in-union women”, where “a union involves a man and a woman regularly cohabiting in a marriage-like relationship”. CPR is “number of women of reproductive age who are married or in a union and who are currently using a method of contraception”, divided by “number of women of reproductive age who are married or in a union”. We used CPR (%) for the period 2006–2010, for women or couples “using any modern method” (defined as including sterilization, IUD, implant, injectable, pill, condom, vaginal barriers, lactational amenorrhea method, emergency contraception, or other, e.g. contraceptive patch or vaginal ring). A limitation is that sexually active unmarried women and those not in unions, e.g. adolescents, are not included in the UN data (for a study including these categories and also traditional contraceptive methods in 77 countries, see [59]). We only included modern methods, as they are most effective and emphasized. Three of the 25 W Europe countries lacked CPR data (Cyprus, Iceland, Luxembourg) but had data for school years, GDP per capita and religiosity. They were included in analyses of these factors.

To examine TFR in relation to FP programs in four regions, we used data on FP program strength ([60] and the website track20.org). Program-effort scores are given for four components (policy, service, records and evaluation, and contraceptive availability and access) and for a total of 30 measures across components (3–13 measures per component, see [60]). A total score is calculated from the component scores. The recommended data to use for countries is this score expressed as a percentage of the maximum score [60]. We used data from 2014, and included only countries that we also used in analyses of the factors above. The number of countries were: Latin America 15, Arab States 9, Sub-Saharan Africa 23 and Asia 12. In a complementary analysis of Sub-Saharan Africa and Asia we used the full sample of countries available at track20.org. We also compared the four regions with respect to their mean program strength in 2014.

### Calculations and statistics

Based on countries, we calculated mean TFR (± SD) and mean values of the other factors for each region. All countries have equal weight in the analyses. In graphs we present the mean regional TFR related to each factor, with the linear least square regression line and the coefficient of determination, *r*^*2*^, for the relationship (e.g. [61]). We similarly explored relationships between TFR and the factors within the six regions, using countries as units. We refrain from statistical testing of regression slopes, as explained above. Outliers and countries at opposite ends of the line are indicated in the graphs (maximum five countries).

School years, GDP per capita, religiosity and contraceptive use may be associated with each other. Their pairwise relationships are shown graphically together with the correlation coefficient *r*. No regression line is shown in these cases, where our purpose is mainly to identify associations among factors, and potential indirect effects on TFR. Estimating influences and dependence/independence among the factors requires other approaches. We summarize this analysis in the Results. Detailed graphs for the six regions, with all countries, are given in Additional file 1 (part 1).

Our main aim here is to analyze variation in TFR. Data on variation in the five factors for all countries are shown in Additional file 1 (part 2).

## Results

### Levels of TFR and related factors in the six regions

**Fig. 1:**
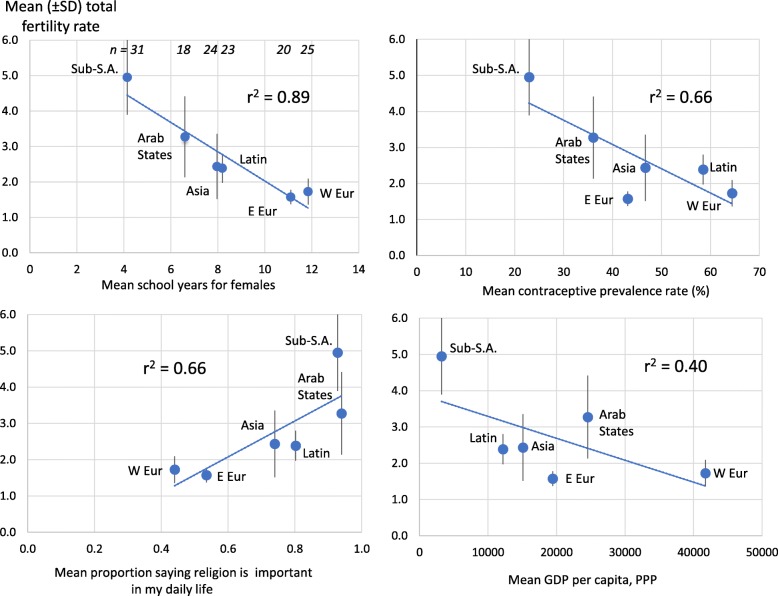
Mean (±SD) Total Fertility Rate (TFR) of Countries in Six Global Regions and its Relationship to Four Factors

E Europe had the lowest TFR (mean 1.57) and Sub-Saharan Africa the highest (4.95). Arab States had the second highest TFR (3.27), 1.7 less than Sub-Saharan Africa. TFR in E Europe (1.57) and W Europe and related countries (1.73) was well below the approximate global replacement rate of 2.1 children per woman. Latin America and Asia had similar TFR: 2.39 and 2.44, respectively. TFR variation within regions was relatively high in Sub-Saharan Africa, Arab States and Asia, lower in Latin America and W Europe, and very low in E Europe (see SD in Fig. 1).

The average number of school years for females varied from 4.2 in Sub-Saharan Africa to 11.8 in W Europe (Fig. 1). TFR declined with increasing school years among the regions (*r*^*2*^ = 0.89). In contrast, TFR increased with religiosity (*r*^*2*^ = 0.66, Fig. 1). The average proportion of respondents saying yes to “Is religion an important part of your daily life?” varied from 0.44 in W Europe to 0.94 in Sub-Saharan Africa.

The average contraceptive prevalence rate (CPR) varied from 23% in Sub-Saharan Africa to 64% in W Europe. TFR was negatively related to CPR (*r*^*2*^ = 0.66) and to GDP per capita (*r*^*2*^ = 0.40, Fig. 1). GDP per capita varied more than ten-fold; it was lowest in Sub-Saharan Africa and highest in W Europe. E Europe deviated most from the regression lines for TFR versus CPR and TFR versus GDP per capita (Fig. 1).

**Fig. 2:**
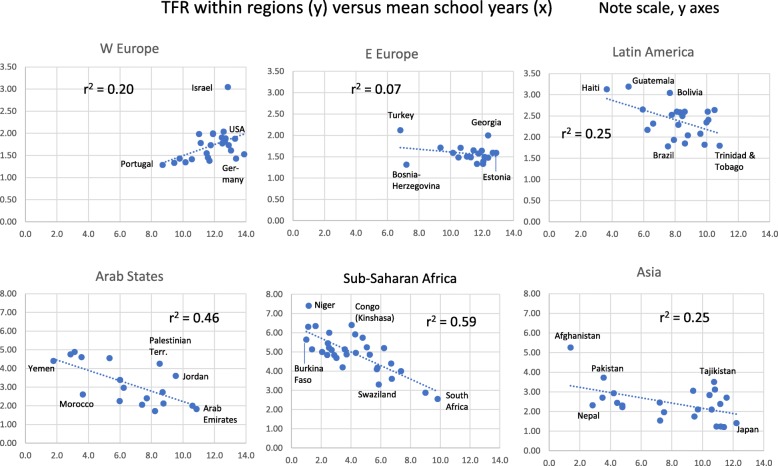
Total Fertility Rate (TFR) for Countries Within each of Six Global Regions, in Relation to Mean Number of School Years for Females (Note Different Scales on Y-axes)

**Fig. 3:**
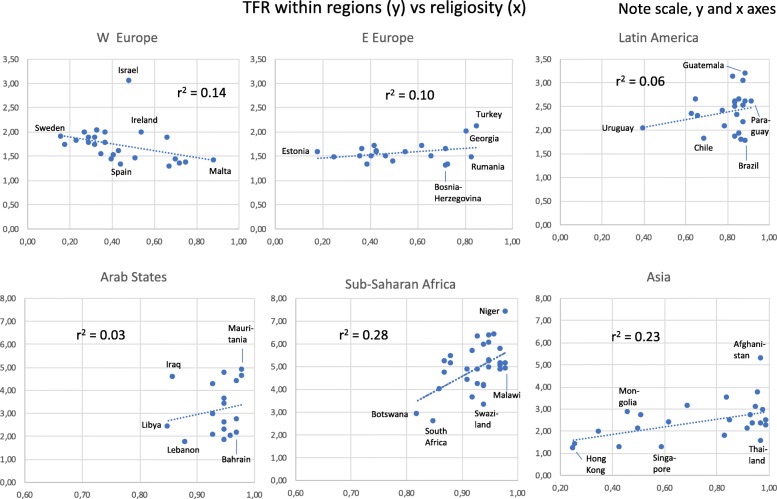
Total Fertility Rate (TFR) for Countries Within each of Six Global Regions, in Relation to the Proportion of Respondents saying “Yes” to the Question “Is Religion an Important Part of Your Daily Life?” (Note Different Scales on Y- and X-axes)

**Fig. 4:**
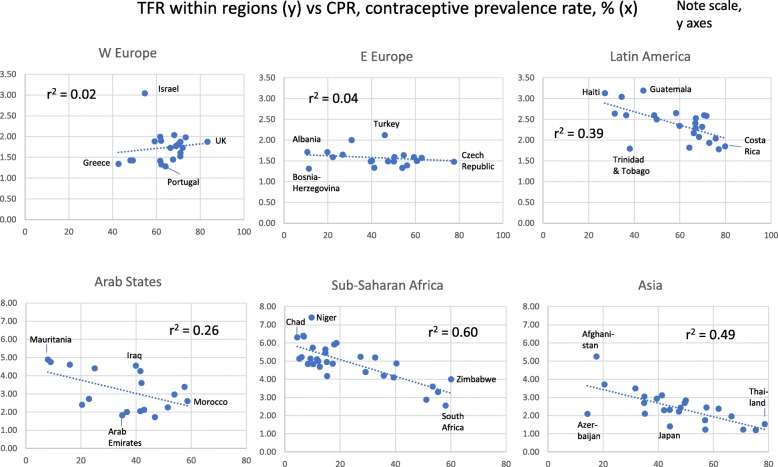
Total Fertility Rate (TFR) for Countries Within each of Six Global Regions, in Relation to Mean Contraceptive Prevalence Rate (%) (Note Different Scales on Y- and X-axes)

**Fig. 5:**
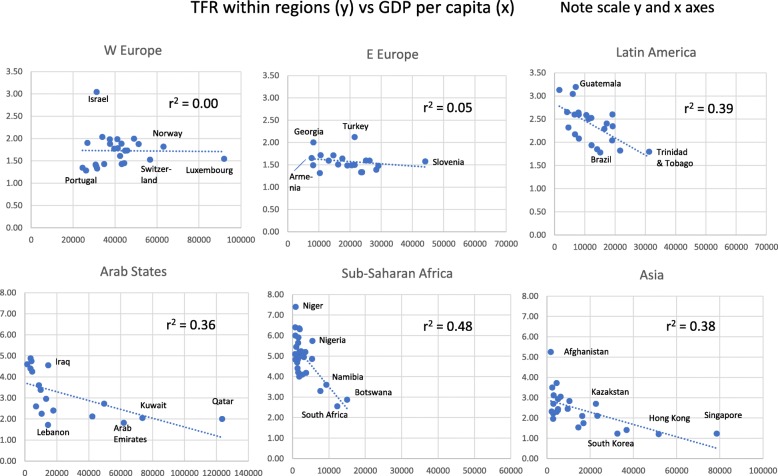
Total Fertility Rate (TFR) for Countries Within each of Six Global Regions, in Relation to GDP Per Capita (international dollars) for the Countries (Note Different Scales on Y- and X-axes)

We also used countries within the regions as units for analysis of TFR’s relation to other factors (Figs. 2, 3, 4 and 5). In five regions TFR decreased with increasing school years (weakest in E Europe, strongest in Sub-Saharan Africa). Surprisingly, it increased with school years in W Europe (Fig. 2). In five regions, TFR increased with religiosity (least strongly in Arab States, strongest in Sub-Saharan Africa and Asia). W Europe deviated again, with negative relation between TFR and religiosity (Fig. 3).

In five regions, TFR had a negative relation to CPR (weak in E Europe, strong in Sub-Saharan Africa and Asia), but a weak positive relation in W Europe (Fig. 4). For GDP per capita, the results were similar: within the regions, TFR decreased with increasing GDP per capita, especially in Sub-Saharan Africa, and also in Latin America, Arab States and Asia, but only weakly so in E Europe, and not at all in W Europe (Fig. 5).

### Relations between the four factors

**Fig. 6:**
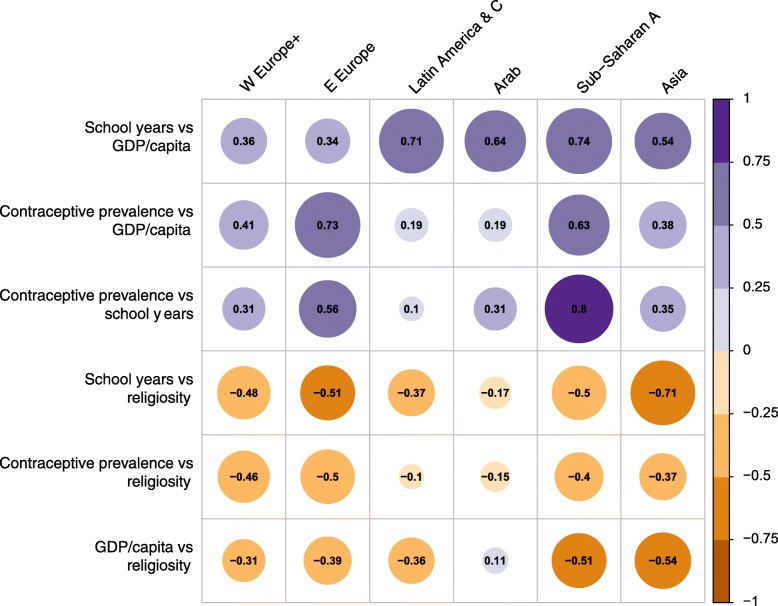
Pairwise Correlations (Pearson’s *r*) Between the Four Factors Related to TFR, with Circle Size Proportional to *r*, and Colors indicating Positive or Negative Correlation

In each region separately, we analyzed the degree to which the factors are correlated (*r*) (for graphs with all countries shown, see Additional file 1, part 1). In W Europe, the three factors associated with TFR decline were positively related (Fig. 6). CPR versus GDP per capita had the strongest correlation, followed by school years versus GDP per capita. Religiosity was negatively correlated with the other factors, most strongly with school years and CPR (Fig. 6).

In E Europe, Latin America and the Arab States the three factors associated with TFR decline also were positively related (Fig. 6), in E Europe most strongly for CPR versus GDP per capita and CPR versus school years, in Latin America and Arab States strongly for school years versus GDP per capita. Religiosity was negatively correlated with the other factors, strongly so for school years. In the Arab states, however, GDP and religiosity were weakly positively related.

In Sub-Saharan Africa the factors were generally more strongly correlated than in other regions (Fig. 6). The three factors associated with TFR decline were positively related, with highest *r* (0.80) between CPR and school years. Religiosity had negative correlations with school years, GDP per capita, and CPR (Fig. 6).

Asia followed the same pattern as the other regions (Fig. 6): the three factors associated with decline in TFR were positively related, especially school years versus GDP per capita. As in W and E Europe, school years reached a maximum of about 12 years for the most affluent countries (see Additional file 1, part 1). Religiosity had strong negative correlation especially with school years, and also with GDP per capita (Fig. 6).

Table 2 gives mean *r* values and their range for the six regions. For factors negatively associated with TFR, the highest mean positive correlation was between school years and GDP per capita. For religiosity, the strongest mean negative correlation was between school years and religiosity (Table 2). Thus, particularly the number of school years for females was correlated with two major factors: positively with GDP per capita, and negatively with religiosity.

**Table 2:** Pairwise Relationships between Factors Negatively Related to TFR (School years, GDP per capita, Contraceptive Prevalence Rate) and between Religiosity, Positively Related to TFR, and the Same Three Factors

Mean *r* Value (Range), *n* = 6 (Regions)		
*Factors Negatively Related to TFR (School years, GDP, CPR)*		
School Years vs GDP per capita	Contraceptive Prevalence Rate (CPR) vs GDP per Capita	Contraceptive Prevalence Rate (CPR) vs School Years
0.56 (0.34–0.74)	0.42 (0.19–0.73)	0.42 (0.10–0.80)
*Factor Positively Related to TRF (Religiosity)*		
School Years vs Religiosity	GDP per Capita vs Religiosity	Contraceptive Prevalence Rate (CPR) vs Religiosity
-0.46 (−0.17, −0.71)	−0.33 (0.11, −0.51)	−0.33 (−0.10, −0.50)

Source: See Methods, for data

### Family planning and TFR

**Fig. 7:**
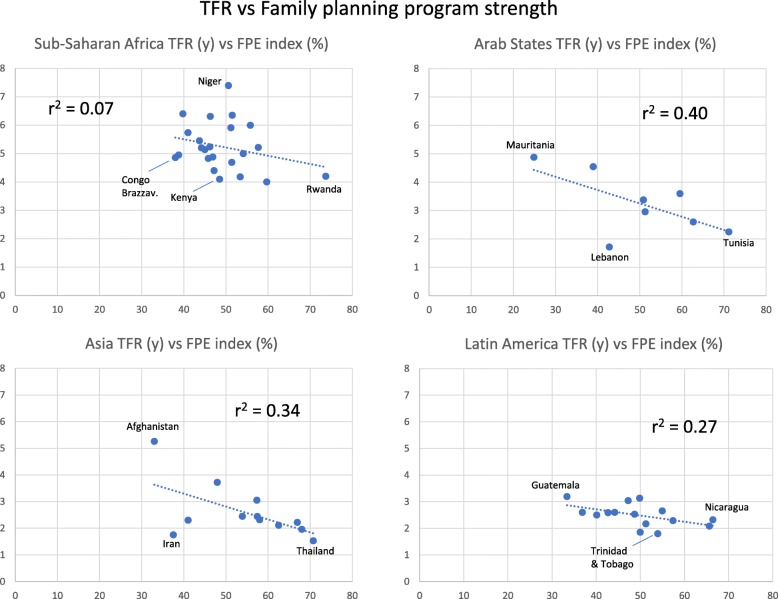
Total Fertility Rate (TFR) for Countries Within each of Four Global Regions, in Relation to Strength of Family Planning Programs (FPE index)

In four regions, we related countries’ TFR to family planning (FP) in 2014. TFR was negatively associated with FP program strength; *r*^2^ ranged from weak (0.07 in Sub-Saharan Africa) to relatively strong relations in the other three regions (0.27–0.40) (Fig. 7). In three regions, *r*^2^ is sensitive to outliers. In Arab States, *r*^2^ changes from 0.40 to 0.85 if the outlier Lebanon is removed. In Asia, *r*^2^ changes from 0.34 to 0.65 if Iran is removed. In contrast, in Sub-Saharan Africa a weak relation becomes even weaker if Rwanda is removed (*r*^2^ changes from 0.07 to 0.02).

In our complementary analysis of Sub-Saharan Africa and Asia, using all countries available at track20.org, the result for Sub-Saharan Africa (*n* = 32) was the same as before (*r*^2^ = 0.07). For Asia (*n* = 27) the correlation TFR versus FP program strength became weaker (*r*^2^ dropped from 0.34 to 0.13). There was high variability of TFR at low program strength, mainly due to the addition of Russia, Armenia, and Azerbaijan.

For the four regions we also re-analyzed TFR versus the other four factors, using the countries in the data set for FP program strength (sample in Fig. 7) and comparing with the earlier result for the full sample of countries (n-values in Fig. 1). For the FP program data set compared to the full sample, *r*^2^ values for the four regions were rather similar for TFR versus school years and CPR, but only about half as large for TFR versus religion and GDP per capita (see Additional file 1, part 3). We also repeated the analysis including only countries deleted from the full sample (those without FP program data, *n* = 38). Analysis of the deleted countries reversed the picture: mean *r*^2^ doubled for TFR versus religion, and TFR versus CPR and GDP per capita also increased markedly, compared to the full sample. For school years, one region had a strongly negative and another region strongly positive relation with TFR (Additional file 1, part 3).

These contrasting results indicate that the FP program dataset was not representative for the full sample of countries. This was confirmed for Asia, Sub-Saharan Africa and Arab States: the countries absent from the FP program analysis (n = 38) had either low or high TFR (and they were included in the full sample). There was a clearly visible gap among TFR values, and the mean values for these countries with low and high TFR were 1.3 and 2.8 for Asia, 3.1 and 5.2 for Sub-Saharan Africa, and 2.2 and 4.5 for Arab States, respectively. Thus, for three regions, several countries with low or high TFR were lacking in the analysis of TFR versus FP program strength (Fig. 7) (Additional file 1, part 3).

Comparing the four regions, the mean FP program strengths in 2014 (based on countries in Fig. 7) were surprisingly similar, ranging only from 49.5 to 54.5%. A complementary analysis with all available countries in track20.org gave an even narrower range, from 48.6 to 52.1% (lowest for Sub-Saharan Africa, highest for Asia). FP program strength therefore was far from a possible 100% maximum value in all four regions.

## Discussion

The broad analysis of six global regions shows associations of TFR with each of the five factors explored (Figs. 1, 2, 3, 4, 5, and 7). The similarity of results among and within regions suggests that the relationships (negative or positive) are real and fairly general. Intriguing deviations occur in W and E Europe. Moreover, the factors to which TFR is related are themselves related in interesting ways, especially female education, which is positively correlated with GDP per capita and negatively correlated with religiosity.

TFR is strongly associated with education, contraceptive use, and religiosity (r^2^ = 0.89, 0.66 and 0.66, respectively). Among regions (Fig. 1), TFR decreased with increasing education for females, supporting earlier studies (e.g. [18, 19, 21, 62]). The number of school years for women increased markedly after 1970 in most regions, but increased less in Africa [63]. The decrease in TFR might also arise indirectly via school year correlations with improved economy, family planning (FP) programs, and media attention to FP, factors which may also lead to smaller families [64–67].

Below, “among regions” refers to comparisons of regions, and “within region” refers to comparisons of countries within regions.

### Western Europe and related countries

Within this region, TFR and education were positively associated, in contrast to all other regions (Fig. 2). This result is consistent with the reversal of TFR decline between 1975 and 2005 in Western countries at high (and increasing) values of the Human Development Index [7, 68, 69]. Also increasing immigration to W Europe may influence TFR (see [70, 71]).

TFR had little or no association with contraceptive prevalence (CPR) or GDP per capita. In contrast to non-European regions, TFR here tended to decline with higher religiosity, partly due to south European countries: among the six countries with highest religiosity, five were in S Europe (Portugal, Italy, Greece, Malta and Cyprus), all with low TFR. But within W European countries, there is evidence that TFR is lowest for religiously unaffiliated or more secular groups [72, 73]. Compared to non-European regions, few European countries have strong religiosity.

### Eastern Europe

E and W Europe had similar average TFR, school years and religiosity, but E Europe had lower CPR and much lower GDP per capita. In contrast to W Europe, TFR in E Europe had no or weak relation to education (Fig. 2). History, post-Soviet economic uncertainty and low GDP per capita may account for higher mean mother’s age at childbirth in E Europe ([74]; see also [75]). Note that CPR measures modern contraceptives, whereas E European methods include high prevalence of withdrawal, rhythm method, and abortion [76]). Contraceptive use in E Europe may therefore be higher than in Fig. 1, and the relation TFR versus CPR among regions stronger than shown there.

Within E Europe there was no or weak relation between TFR and GDP per capita (Fig. 5), but note that TFR varies little. Education had high levels in both E and W Europe. Hilevych & Rusterholz [77] suggested that female labor force participation and contraceptive use favor small families (low TFR) in both E and W Europe. In addition, countries in these two regions may have gone through a ‘second demographic transition’, with a diversity of union and family types and very low TFR (see [78], and review in [79]).

### Latin America and the Caribbean

Among regions, Latin America and Asia are intermediate in TFR level and religiosity. Latin America had the second lowest GDP per capita and, perhaps surprisingly, the second highest CPR. In many countries, such as Chile, Colombia, Costa Rica and Mexico, family planning activities, policies or programs started and expanded in the 1960’s and 1970’s. Despite resistance from the Vatican, modern contraception became widespread early [30, 39, 80].

Within Latin America TFR declined with more education, but it declined more strongly with increased CPR and GDP per capita (Figs. 4, and 5), suggesting that these factors may be more important than education for TFR in Latin America. School years and GDP per capita were strongly positively associated, suggesting that economic resources sometimes limit education. CPR on the other hand was weakly related to GDP and education, and may partly be limited by other factors – possibly religiosity, through its negative correlation with education. At higher levels of religiosity in Latin America (proportion > 0.8) there is remarkable variation in school years and CPR among countries. At high levels of religiosity, some countries therefore achieve high levels of female education and CPR, in contrast with others at similarly high level of religiosity. This variation deserves further study, see Additional file 1 (part 1).

### Arab states

Arab States had the second highest TFR among the regions, low CPR, and an unusual combination of highest religiosity and second highest GDP per capita among the regions. In some countries, oil resources have led to wealth, but the mean for female school years is low (very low for some countries). Within the region TFR declined strongly with increased education, GDP per capita, and CPR. TFR and religiosity were weakly associated, but note the small variation: almost all countries are highly religious.

The Arab States began implementing FP programs fairly recently, during the 1990’s ([81]; for exceptions, such as Tunisia and Morocco, see [39]). Effects of FP efforts may come in the future, unless religiosity hinders TFR decline ([63], and references therein). As in Latin America, at high levels of religiosity (proportion > 0.9) there is large variation in school years, GDP, and CPR among the countries. Arab State social norms, also associated with religion, generally disfavor female empowerment [82].

### Sub-Saharan Africa

This region stands out with much higher TFR and markedly lower CPR than in the other five regions. The level of religiosity is high, similar to Arab States, but GDP per capita is much lower. Within Sub-Saharan Africa, TFR is strikingly negatively correlated with education, GDP and CPR, which all may affect TFR. Two ‘natural experiments’, involving changes in schooling in Nigeria [83] and Uganda [84], support the role of education for TFR. School years, GDP and CPR were strongly positively correlated, particularly CPR and school years, suggesting that education favors contraceptive use.

Religious influence may be one contributing reason for high TFR, and for stalling TFR decline in this region. For the eight countries with religiosity above 0.95, females had on average only 1–5 school years. Religiosity was considered an important determinant of fertility in Sub-Saharan Africa by e.g. Caldwell & Caldwell [85], Akintunde et al. [35] and Agadjanian & Yabiku [86]. A related and probably strong influence is persistent patriarchal social structure and gender inequality (e.g. [87]). For Burkina Faso, Mali, Niger and Chad, “One of the key barriers to having desired number of children is sociocultural norms, especially the husband’s role as primary decision-maker and the desire for a large family” [88].

### Asia

Among regions, Asia resembled Latin America in TFR, GDP per capita and religiosity, though with lower average CPR (Fig. 1). Within Asia, lower TFR was associated with longer female education and higher GDP, and especially with higher CPR. As in Latin America, several countries with TFR below replacement level had CPR values above 70% (Thailand, South Korea and Hong Kong). FP programs have been important historically in these and other Asian countries [39]. In central Asia, however, Pakistan, Tajikistan and Afghanistan had TFR above 3.5 and low levels of CPR. An interesting exception in central Asia is Azerbaijan, with the lowest CPR (Fig. 4) but with TFR at 2.1. Many female school years (10.6), low religiosity (proportion 0.5), use of traditional contraception [59] and economic conditions [75] may together explain this exception.

The Asian countries show a rather strong positive correlation between education and GDP, and an even stronger negative association between education and religiosity.

### Role of different factors

To help clarify factors of likely importance for TFR in different global regions, we studied five potential major agents that could be quantified. Social norms are also important [89] but often difficult to quantify. For example, large desired family size characterizes Sub-Saharan Africa. Korotayev et al. [49] related this norm to polygyny, high status of polygynous men, extended families, and child fosterage within kinships. The latter two aspects enable females to carry out traditional hoe agriculture without reducing the number of children, contributing to high TFR. And in modern urban Africa, abolition of postpartum sex taboos reduces birth intervals and may contribute to high TFR when large desired family size persists [49, 90, 91].

To limit the number of factors and relationships we did not analyze infant and child mortality, gender roles and female labor force participation rates, which may all play a role [9, 25, 92–96]. These factors seem likely to bear some relation to female education, contraceptive use and GDP per capita. Family planning programs include contraception and education directly related to fertility, and was analyzed in four regions. Lower TFR was associated with stronger FP programs in Asia, Arab States and Latin America, but only weakly so in Sub-Saharan Africa. In a study of 40 countries 2003–2010, TFR levels “were lowest in the presence of both good social settings and strong programs”, but Sub-Saharan Africa was the least successful region ([97], based on data from track20.org). Yet, in 2014, the mean values for program strength were similar in all four regions in our study. However, FP programs in Asia and Latin America started earlier, and many of them are considered successful ([66], and references therein). Duration, change in social norms, institutional support and international funding are important for success of FP programs [27, 40, 46].

Lower TFR was associated with higher FP program strength in three regions. For Sub-Saharan Africa, Arab States and Asia, FP programs were under-represented in low and high TFR countries, compared to our full sample of countries. Incentives for starting FP programs may be lower in countries with relatively low TFR. And such programs might be difficult to start in poor, high-TFR countries with strong religion, corruption or conflicts. Nevertheless, the results in Fig. 7 suggest that FP programs recently have been effective also within relatively narrow TFR ranges in Asia and Arab states, but not in Sub-Saharan Africa.

Among regions, the TFR versus GDP per capita relationship was the weakest of the four (Fig. 1). Without Sub-Saharan Africa, the slope of the regression would be near zero. But within four regions, TFR’s negative relation to GDP per capita was strong or relatively strong (Fig. 5). So why is TFR not associated with GDP per capita in E and W Europe, in line with economic hypotheses, and despite equally large variation in GDP per capita as in Latin America? And why are school years, potentially improving child ‘quality’, not negatively associated with TFR in E and W Europe? The relation is even reversed, TFR increasing with school years in W Europe.

Evidence for a quantity-quality trade-off, between increased family size and investment in child quality, is mixed ([98], and references therein). In India, trade-off was strongest in rural areas [98]. In this study, TFR declined with increasing GDP per capita especially in the three poorest regions (Sub-Saharan Africa, Asia, Latin America). Is there a self-reinforcing loop, where increased wealth motivates higher child quality and other changes that reduce TFR, the reduction feeding back positively on economic development and wealth? According to Canning & Schultz [41], TFR declines can boost income per capita through reduced youth dependency rates, and may have positive long-term economic effects (see also [27–29]).

This study is, as far as we know, the first to relate TFR to religiosity together with other major factors in global regions and many countries. Both among the regions (Fig. 1) and within two of them (Asia and Sub-Saharan Africa, Fig. 3), TFR increased with degree of religiosity. Moreover, stronger religiosity is associated with lower education, CPR and GDP per capita in at least five regions. Among Arab States, effects of the large differences in wealth seem to override effects of the small differences in strength of religiosity.

We quantified religiosity from Gallup surveys, but did not distinguish between religions as regard TFR. There are probably differences [37, 86, 99], but using the same basic measure greatly simplifies regional and global analyses. In a study in the US, religiosity measured as here was more useful than religious affiliation and showed “a substantially positive effect on fertility”, without any gender difference [100]. Most earlier studies analyzed religious affiliation and TFR. Global TFR 2010–2015 was substantially lower for religiously non-affiliated (1.7) than for affiliated (2.6) [36].

Why is fertility associated with religiosity? Beside declarations from the Vatican and other religious leaders [30, 31], possible reasons are belief in supernatural influence on things we desire, such as “good crops, protection, health and fertility” [33, 101], and fatalistic views about fertility, such as children “are up to God” [46, 89]. Human sociality and norms, history, type of religion and other conditions influence TFR-religion relationships [86, 99, 102]. Religiosity probably contributes to maintaining high TFR in Sub-Saharan Africa, Arab States and parts of Asia and Latin America, in part by suppressing factors that reduce TFR. Yet FP programs have been successful even in strongly religious countries, as shown by encouraging results in Iran [103], Tunisia [104], and Rwanda [90].

## Conclusions

Total fertility rate (TFR) is lower with longer average education for females, higher GDP per capita, higher contraceptive prevalence rate, and stronger family planning programs. These recent relations hold generally among regions, but less so in E Europe, and not at all in W Europe and related countries. In contrast, TFR is higher when religiosity is stronger. Religiosity is also associated with fewer school years, lower GDP per capita and less contraceptive use, in line with several studies of religion, gender aspects, and socioeconomic development ([105, 106], but see also [30]).

To clarify causality, further studies of TFR in relation to these five and other potentially important factors are needed. Longitudinal studies and controlled or ‘natural’ experiments [41] are valuable, but studies of current conditions are also desirable for TFR policy decisions. More studies are needed of how FP programs started and progressed in different countries and religious settings. The role of media in changing gender norms and contraceptive use also needs further study [66, 107].

Human fertility rate has critical consequences for the entire biosphere [2, 6, 12, 108], but conclusions about the main factors that determine TFR vary markedly between researchers (see for instance [8, 18, 66]). Lack of consensus calls for more research on the importance of e.g. content and quality of education (as pointed out by Cleland, [109]). Norris & Inglehart [34] remarked that “the world as a whole is becoming more religious” (see also [32, 33]). The role of religiosity therefore needs more study; it might be involved in stalling TFR decline in several countries.

## Supplementary information

**Additional file 1.** Relation among factors (part 1), variation in factors (part 2), and family planning dataset (part 3).

## Data Availability

All data analysed during this study are included in published article and Supplementary Information; sources for the data are available to anyone freely on the Internet, or in published books, by Reference list.

## Abbreviations

CPR: Contraceptive Prevalence Rate
FP: Family Planning
GDP: Gross Domestic Product
TFR: Total Fertility Rate
UN: United Nations
