# Supplementary material for: Human fertility in relation to education, economy, religion, contraception, and family planning programs
Source: BMC Public Health. 2020 Feb 22;20:265. doi: 10.1186/s12889-020-8331-7 (PMC7036237; doi:10.1186/s12889-020-8331-7)
Supplement: Supplementary file 1 — Additional file 1. Relation among factors (part 1), variation in factors (part 2), and family planning dataset (part 3). [file 12889_2020_8331_MOESM1_ESM.pptx]

## Slide 1
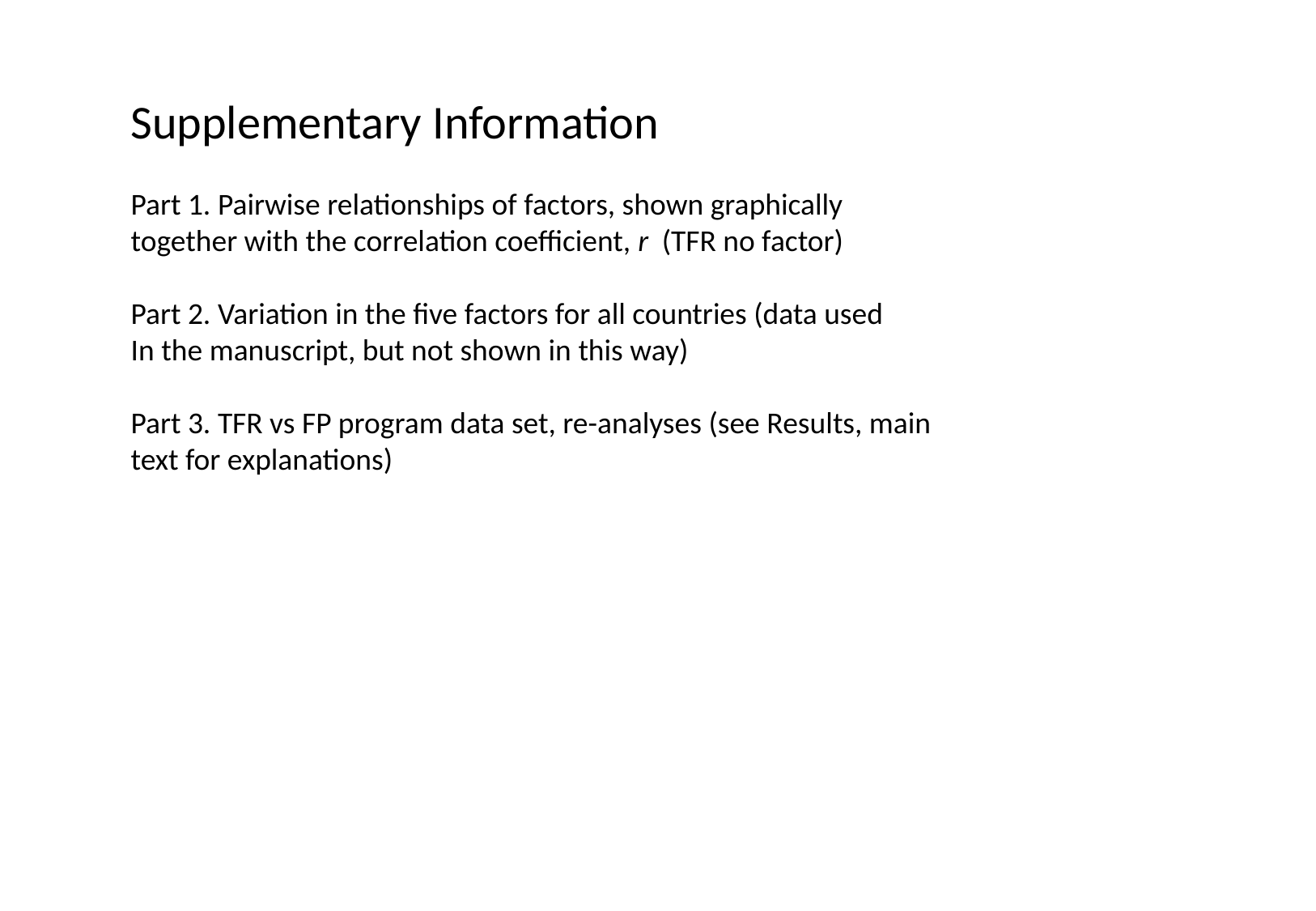

Supplementary Information
Part 1. Pairwise relationships of factors, shown graphically
together with the correlation coefficient, r (TFR no factor)
Part 2. Variation in the five factors for all countries (data used
In the manuscript, but not shown in this way)
Part 3. TFR vs FP program data set, re-analyses (see Results, main
text for explanations)

## Slide 2
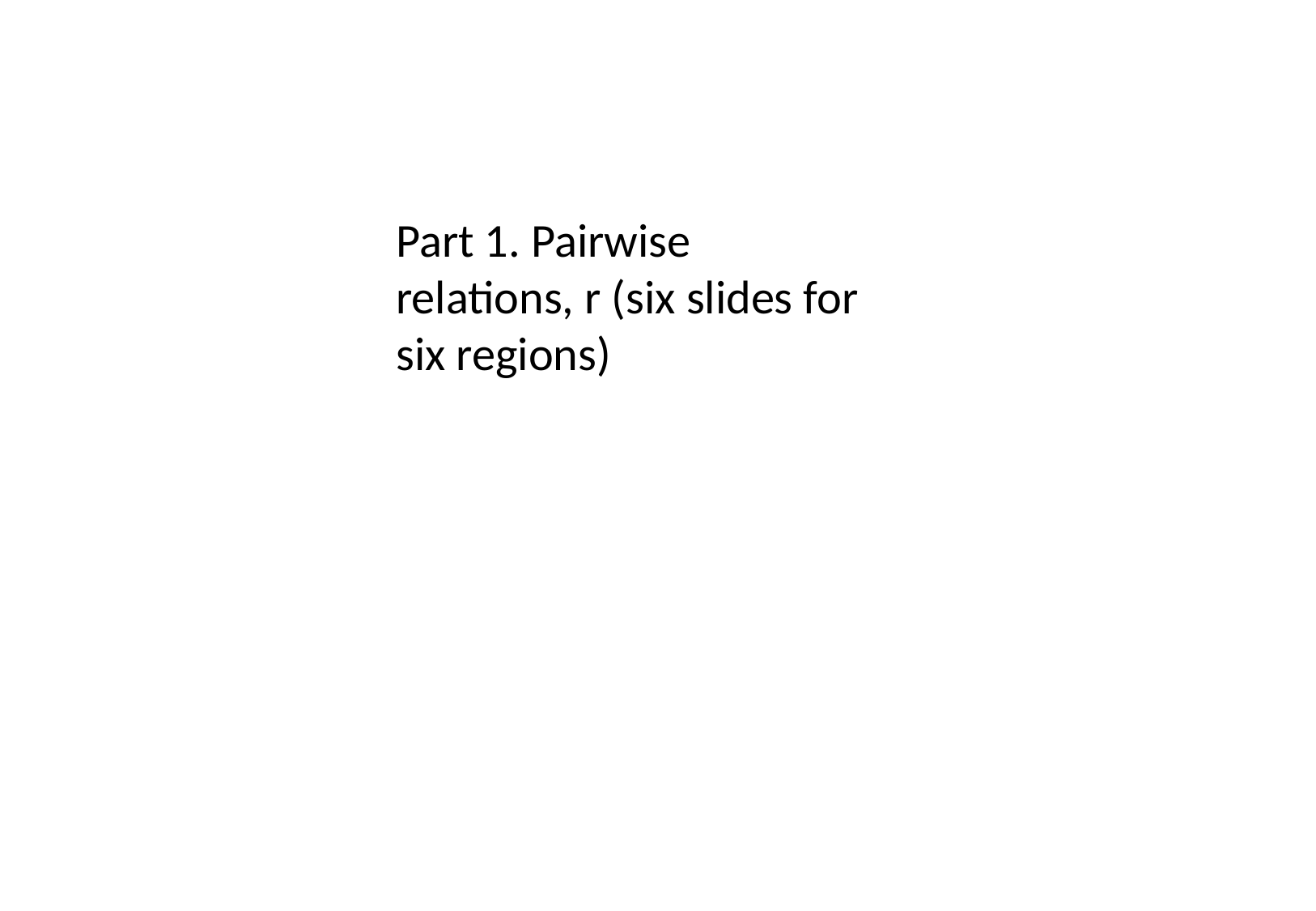

Part 1. Pairwise relations, r (six slides for
six regions)

## Slide 3
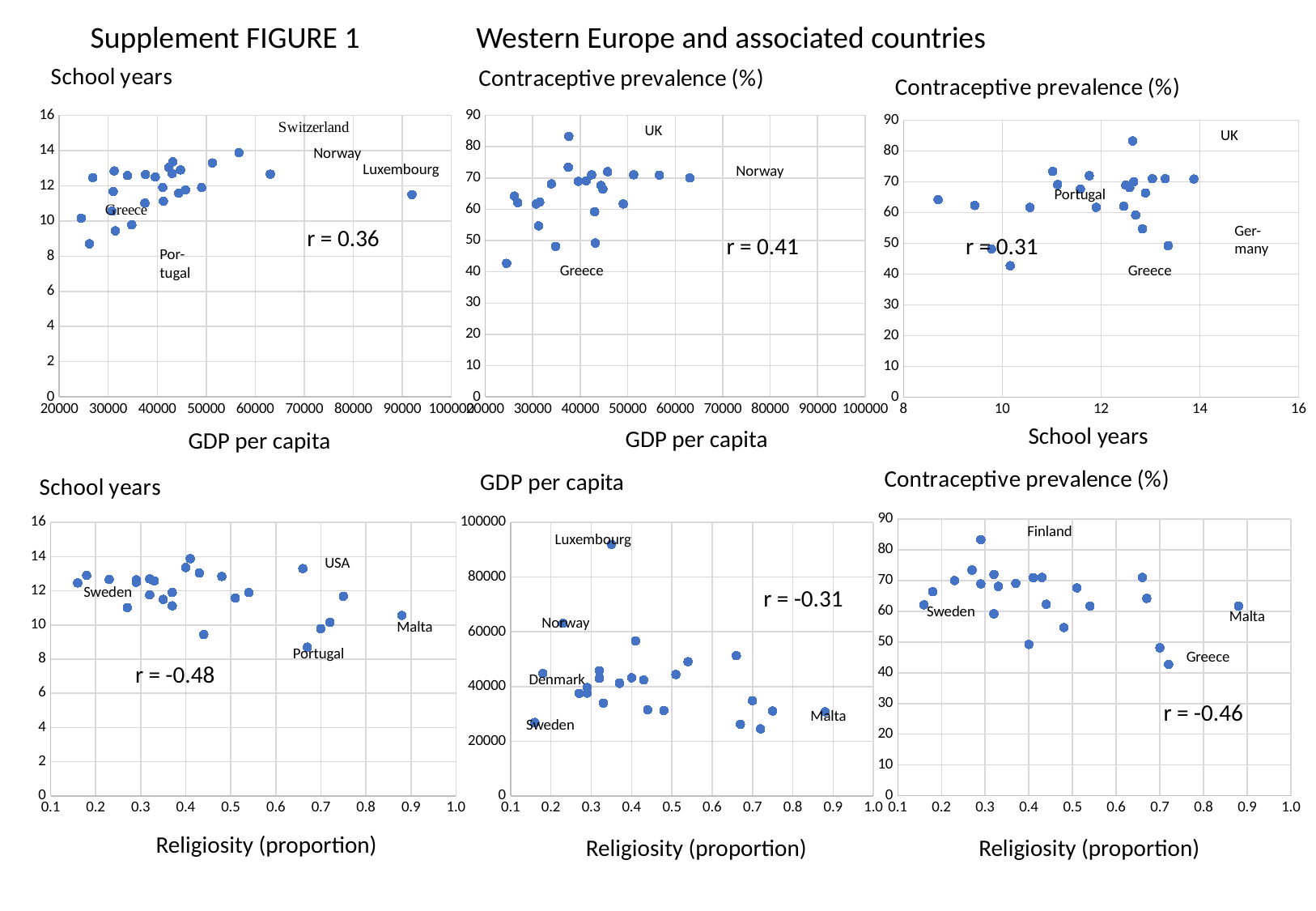

Supplement FIGURE 1
Western Europe and associated countries
### Chart: School years
| Category | |
|---|---|
### Chart: Contraceptive prevalence (%)
| Category | |
|---|---|UK
UK
Norway
Luxembourg
Norway
Portugal
Ger-
many
r = 0.36
r = 0.41
r = 0.31
Por-
tugal
Greece
Greece
School years
GDP per capita
GDP per capita
### Chart: GDP per capita
| Category | |
|---|---|Finland
Luxembourg
USA
Sweden
r = -0.31
Sweden
Malta
Norway
Malta
Portugal
Greece
r = -0.48
Denmark
r = -0.46
Malta
Sweden
Religiosity (proportion)
Religiosity (proportion)
Religiosity (proportion)
### Chart: Contraceptive prevalence (%)
| Category | |
|---|---|
### Chart: Contraceptive prevalence (%)
| Category | |
|---|---|
### Chart: School years
| Category | |
|---|---|

## Slide 4
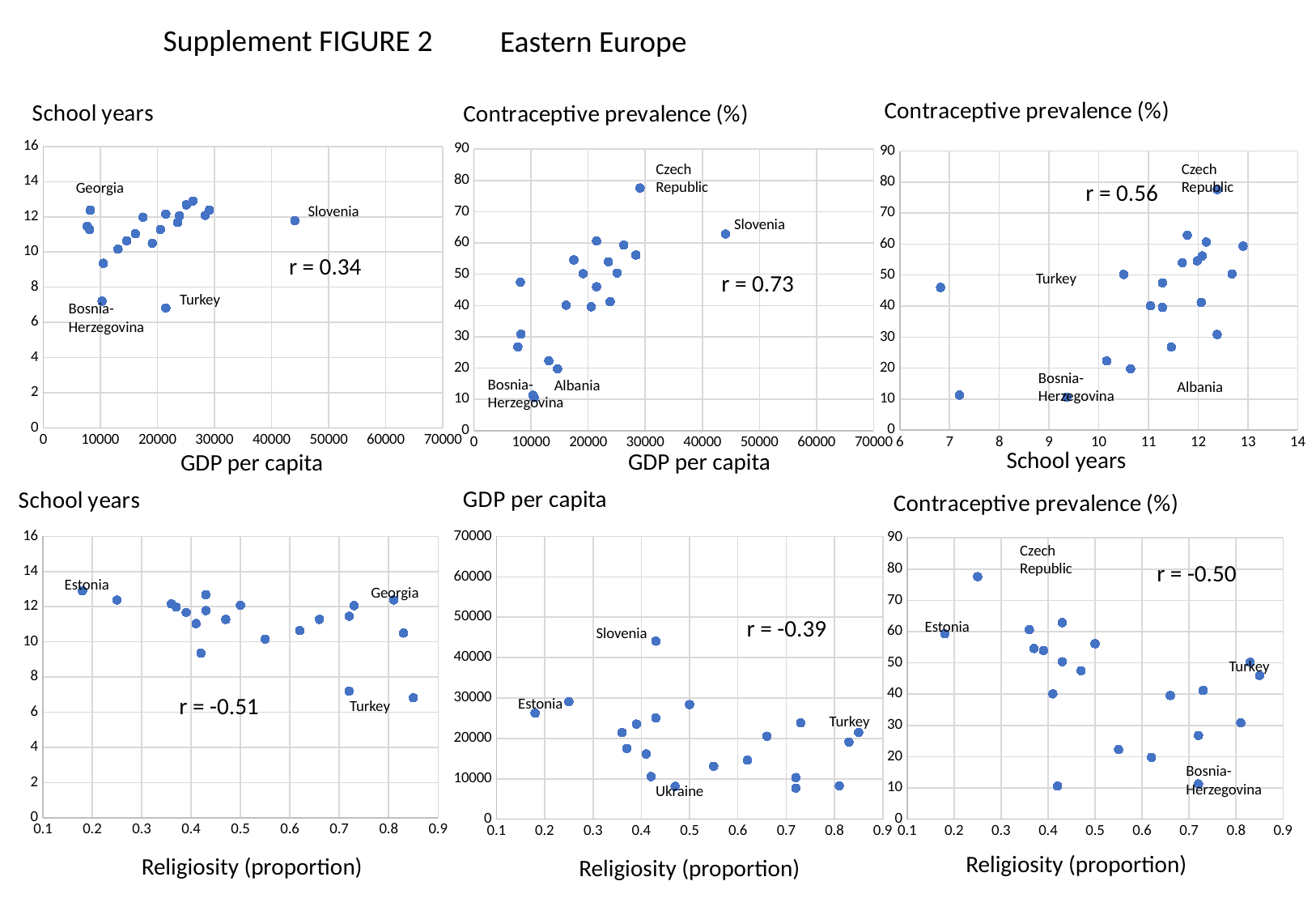

Supplement FIGURE 2
Eastern Europe
### Chart: School years
| Category | |
|---|---|
### Chart: Contraceptive prevalence (%)
| Category | |
|---|---|Czech
Republic
Czech
Republic
r = 0.56
Georgia
Slovenia
Slovenia
r = 0.34
r = 0.73
Turkey
Turkey
Bosnia-
Herzegovina
Bosnia-
Herzegovina
Bosnia-
Herzegovina
Albania
Albania
School years
GDP per capita
GDP per capita
### Chart: School years
| Category | |
|---|---|
### Chart: GDP per capita
| Category | |
|---|---|
### Chart: Contraceptive prevalence (%)
| Category | |
|---|---|Czech
Republic
r = -0.50
Estonia
Georgia
r = -0.39
Estonia
Slovenia
Turkey
r = -0.51
Estonia
Turkey
Turkey
Bosnia-
Herzegovina
Ukraine
Religiosity (proportion)
Religiosity (proportion)
Religiosity (proportion)
### Chart: Contraceptive prevalence (%)
| Category | |
|---|---|

## Slide 5
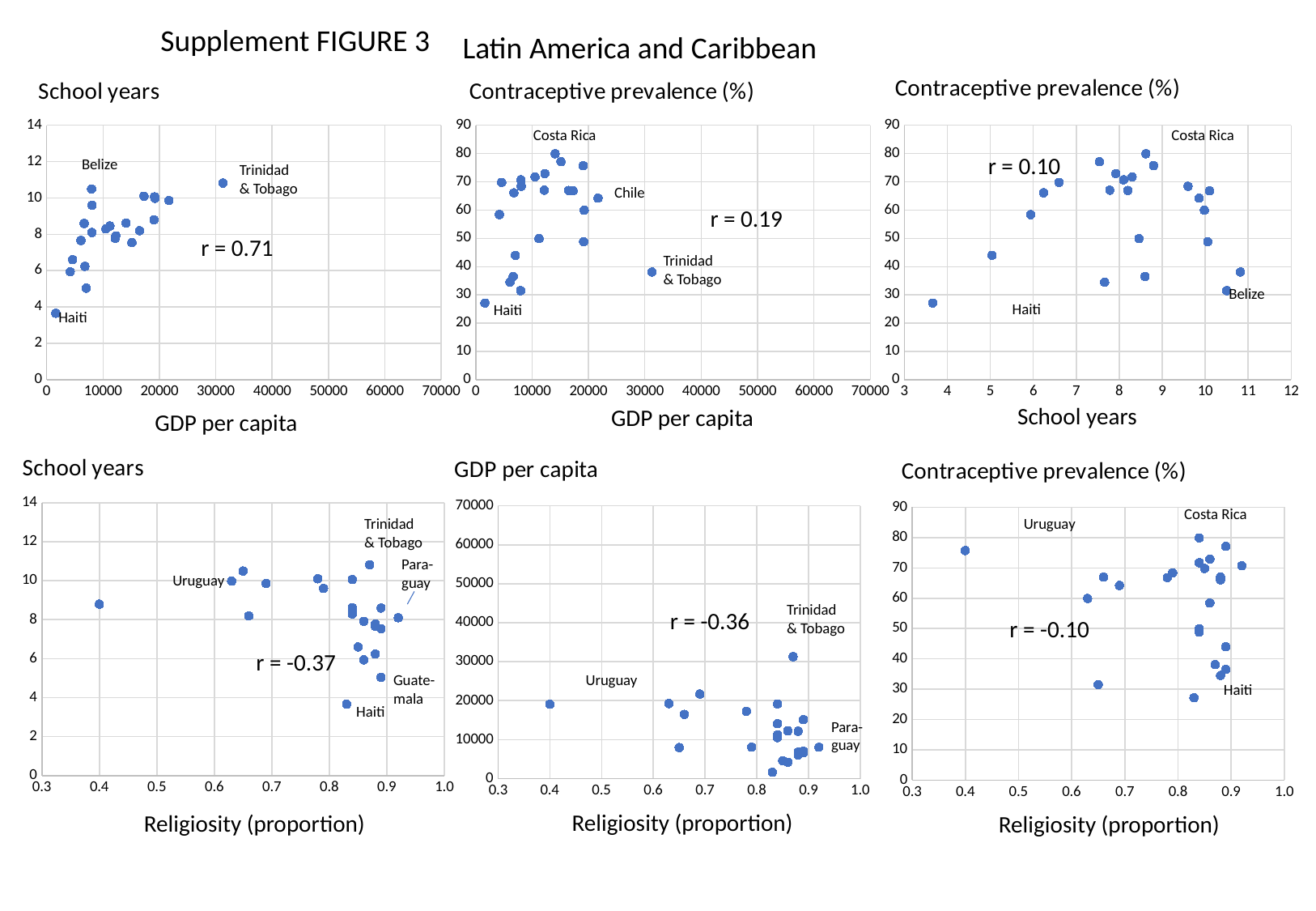

Supplement FIGURE 3
Latin America and Caribbean
### Chart: School years
| Category | |
|---|---|
### Chart: Contraceptive prevalence (%)
| Category | |
|---|---|Costa Rica
Costa Rica
r = 0.10
Belize
Trinidad
& Tobago
Chile
r = 0.19
r = 0.71
Trinidad
& Tobago
Belize
Haiti
Haiti
Haiti
School years
GDP per capita
GDP per capita
### Chart: GDP per capita
| Category | |
|---|---|Costa Rica
Trinidad
& Tobago
Uruguay
Para-
guay
Uruguay
Trinidad
& Tobago
r = -0.36
r = -0.10
r = -0.37
Uruguay
Guate-
mala
Haiti
Haiti
Para-
guay
Religiosity (proportion)
Religiosity (proportion)
Religiosity (proportion)
### Chart: Contraceptive prevalence (%)
| Category | |
|---|---|
### Chart: School years
| Category | |
|---|---|
### Chart: Contraceptive prevalence (%)
| Category | |
|---|---|

## Slide 6
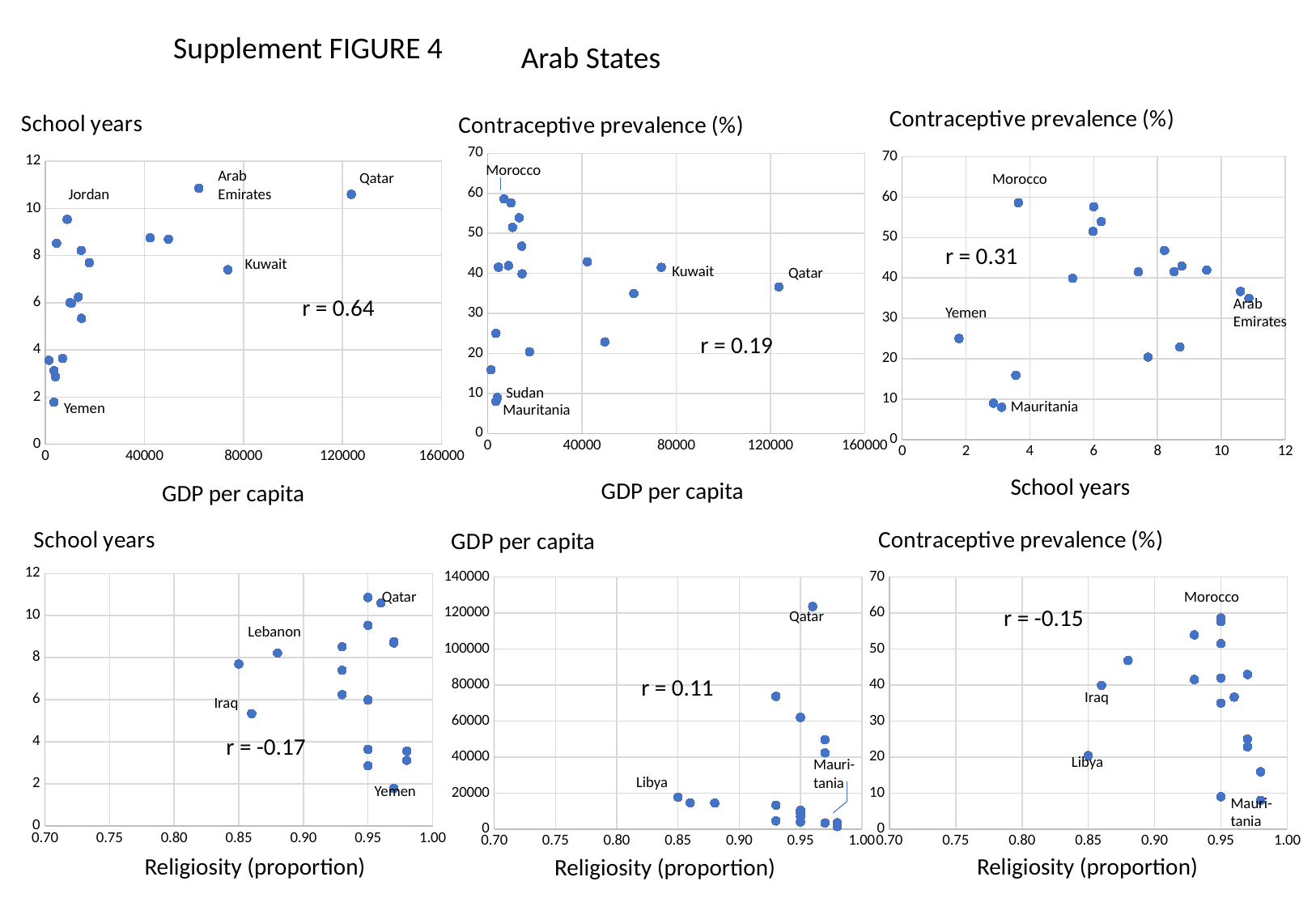

Supplement FIGURE 4
Arab States
### Chart: Contraceptive prevalence (%)
| Category | |
|---|---|
### Chart: Contraceptive prevalence (%)
| Category | |
|---|---|
### Chart: School years
| Category | |
|---|---|Morocco
Arab
Emirates
Qatar
Morocco
Jordan
r = 0.31
Kuwait
Kuwait
Qatar
r = 0.64
Arab
Emirates
Yemen
r = 0.19
Sudan
Mauritania
Yemen
Mauritania
School years
GDP per capita
GDP per capita
### Chart: School years
| Category | |
|---|---|
### Chart: GDP per capita
| Category | |
|---|---|Qatar
Morocco
r = -0.15
Qatar
Lebanon
r = 0.11
Iraq
Iraq
r = -0.17
Libya
Mauri-
tania
Libya
Yemen
Mauri-
tania
Religiosity (proportion)
Religiosity (proportion)
Religiosity (proportion)
### Chart: Contraceptive prevalence (%)
| Category | |
|---|---|

## Slide 7
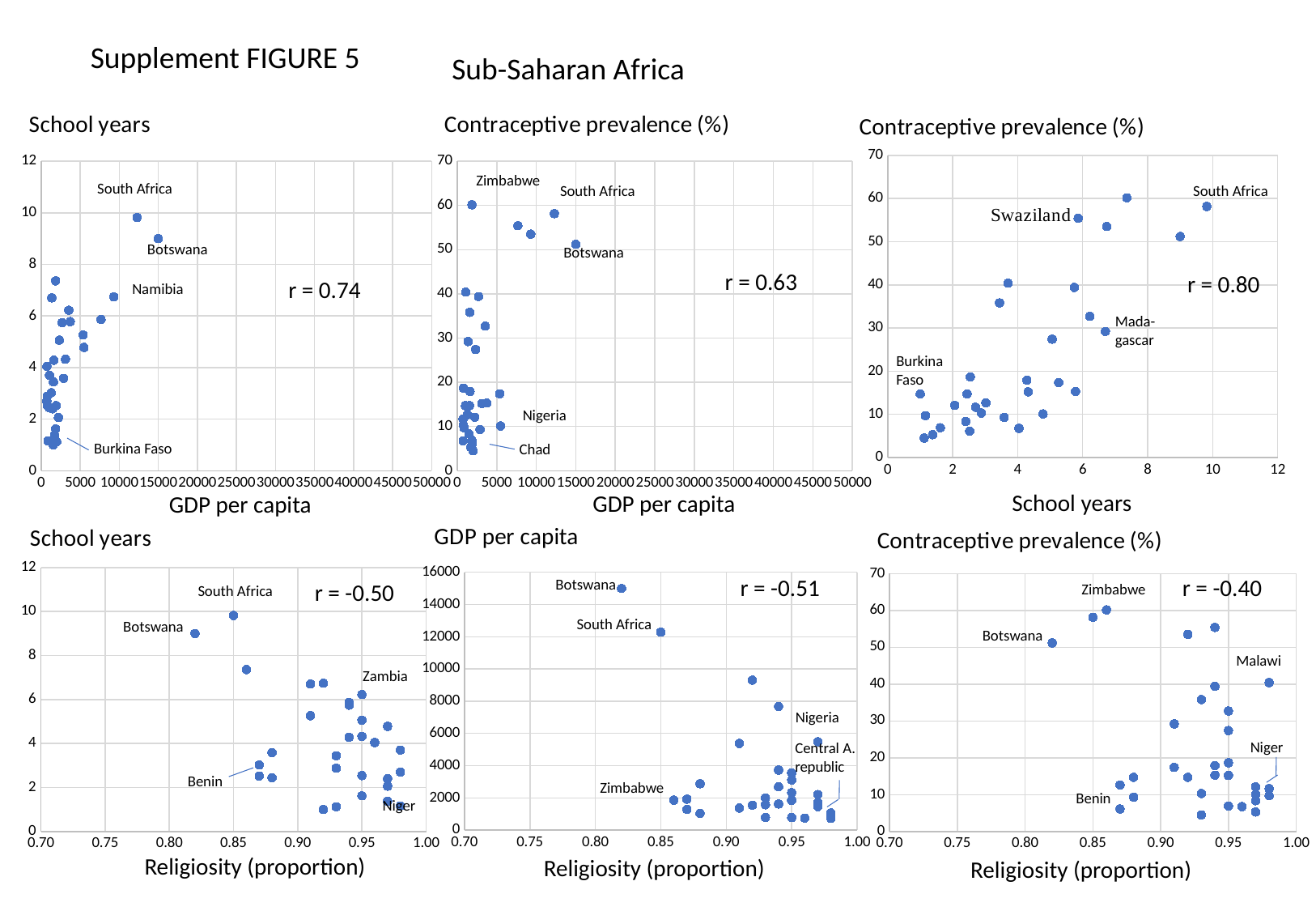

Supplement FIGURE 5
Sub-Saharan Africa
### Chart: School years
| Category | |
|---|---|
### Chart: Contraceptive prevalence (%)
| Category | |
|---|---|Zimbabwe
South Africa
South Africa
South Africa
Botswana
Botswana
r = 0.63
r = 0.80
r = 0.74
Namibia
Mada-
gascar
Burkina
Faso
Nigeria
Burkina Faso
Chad
School years
GDP per capita
GDP per capita
### Chart: School years
| Category | |
|---|---|
### Chart: GDP per capita
| Category | |
|---|---|r = -0.40
r = -0.51
Botswana
r = -0.50
Zimbabwe
South Africa
South Africa
Botswana
Botswana
Malawi
Zambia
Nigeria
Niger
Central A.
republic
Benin
Zimbabwe
Benin
Niger
Religiosity (proportion)
Religiosity (proportion)
Religiosity (proportion)
### Chart: Contraceptive prevalence (%)
| Category | |
|---|---|
### Chart: Contraceptive prevalence (%)
| Category | |
|---|---|

## Slide 8
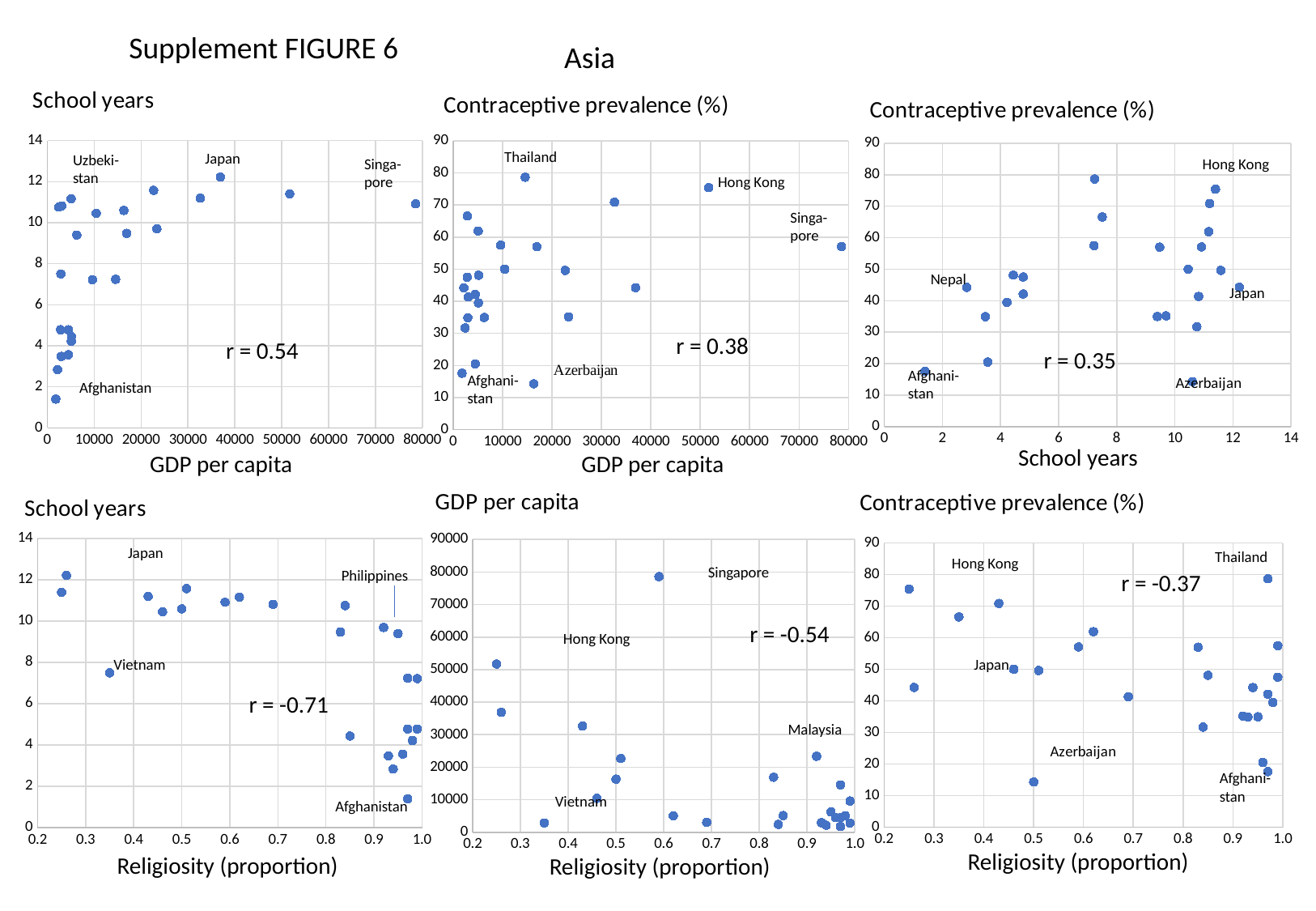

Supplement FIGURE 6
Asia
### Chart: School years
| Category | |
|---|---|
### Chart: Contraceptive prevalence (%)
| Category | |
|---|---|Thailand
Japan
Uzbeki-
stan
Singa-
pore
Hong Kong
Hong Kong
Singa-
pore
Nepal
Japan
r = 0.38
r = 0.54
r = 0.35
Afghani-
stan
Afghani-
stan
Afghanistan
School years
GDP per capita
GDP per capita
### Chart: School years
| Category | |
|---|---|
### Chart: GDP per capita
| Category | |
|---|---|Japan
Thailand
Hong Kong
Singapore
Philippines
r = -0.37
r = -0.54
Hong Kong
Japan
Vietnam
r = -0.71
Malaysia
Afghani-
stan
Vietnam
Afghanistan
Religiosity (proportion)
Religiosity (proportion)
Religiosity (proportion)
### Chart: Contraceptive prevalence (%)
| Category | |
|---|---|
### Chart: Contraceptive prevalence (%)
| Category | |
|---|---|

## Slide 9
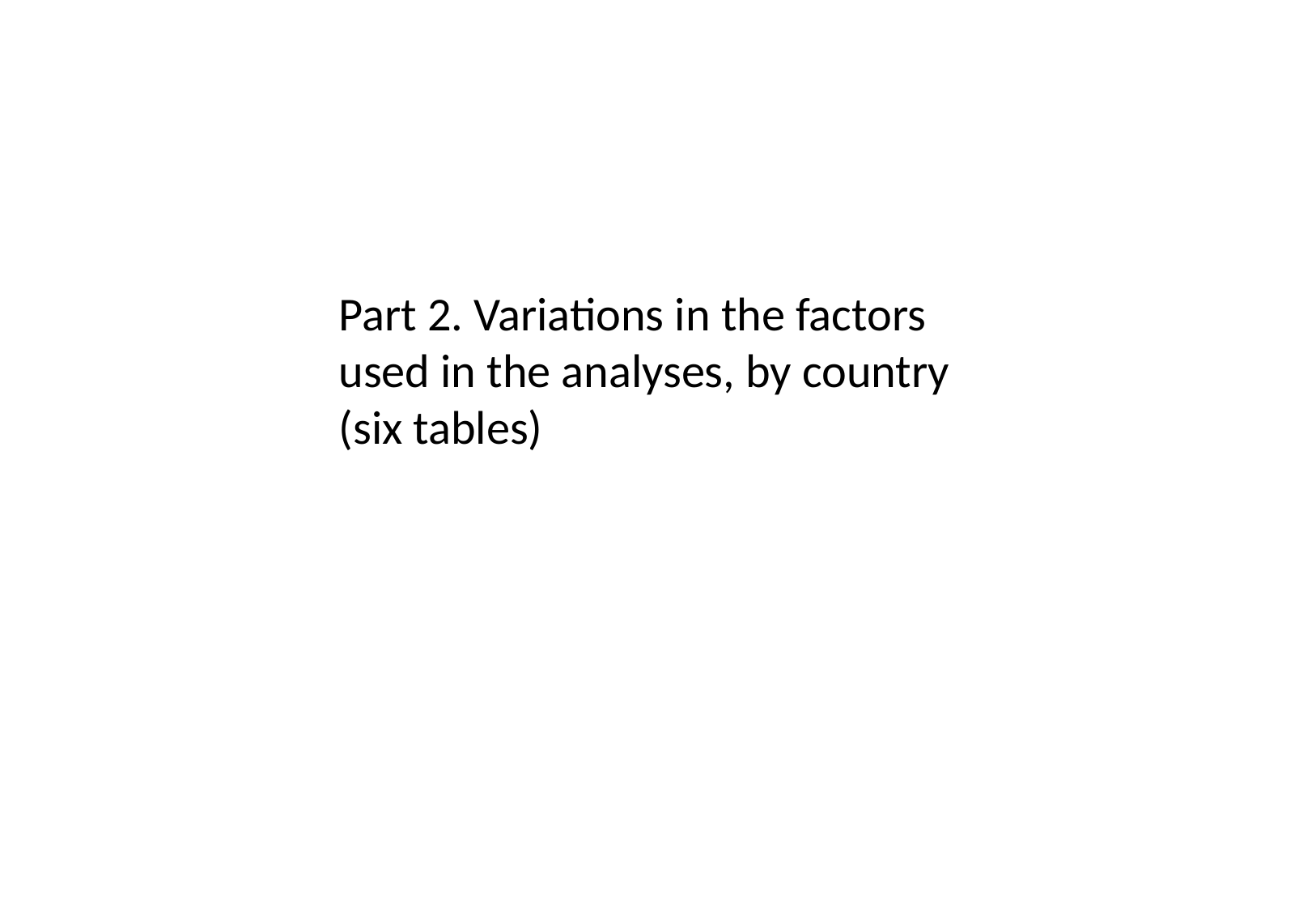

Part 2. Variations in the factors
used in the analyses, by country
(six tables)

## Slide 10
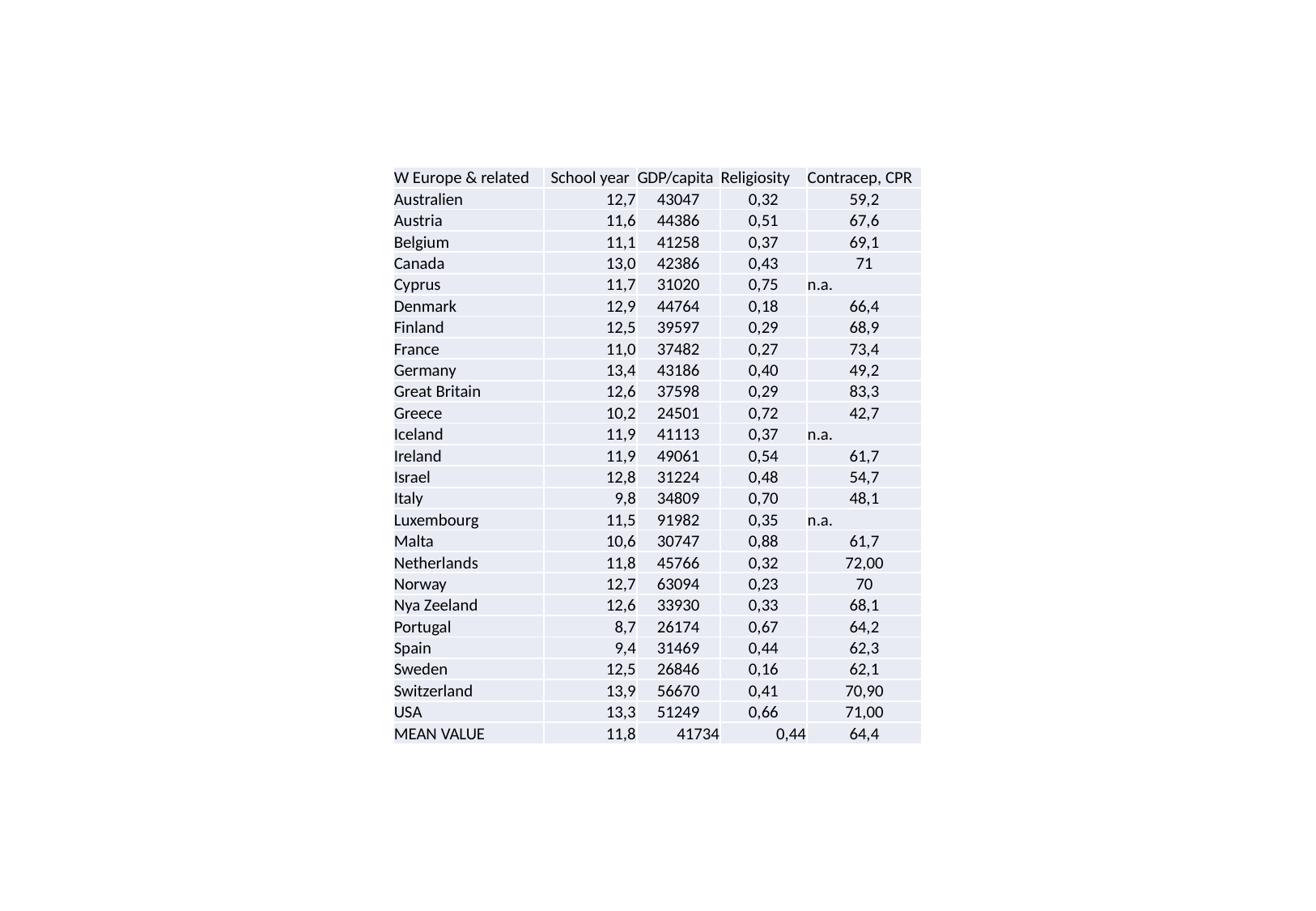

| W Europe & related | School year | GDP/capita | Religiosity | Contracep, CPR |
| --- | --- | --- | --- | --- |
| Australien | 12,7 | 43047 | 0,32 | 59,2 |
| Austria | 11,6 | 44386 | 0,51 | 67,6 |
| Belgium | 11,1 | 41258 | 0,37 | 69,1 |
| Canada | 13,0 | 42386 | 0,43 | 71 |
| Cyprus | 11,7 | 31020 | 0,75 | n.a. |
| Denmark | 12,9 | 44764 | 0,18 | 66,4 |
| Finland | 12,5 | 39597 | 0,29 | 68,9 |
| France | 11,0 | 37482 | 0,27 | 73,4 |
| Germany | 13,4 | 43186 | 0,40 | 49,2 |
| Great Britain | 12,6 | 37598 | 0,29 | 83,3 |
| Greece | 10,2 | 24501 | 0,72 | 42,7 |
| Iceland | 11,9 | 41113 | 0,37 | n.a. |
| Ireland | 11,9 | 49061 | 0,54 | 61,7 |
| Israel | 12,8 | 31224 | 0,48 | 54,7 |
| Italy | 9,8 | 34809 | 0,70 | 48,1 |
| Luxembourg | 11,5 | 91982 | 0,35 | n.a. |
| Malta | 10,6 | 30747 | 0,88 | 61,7 |
| Netherlands | 11,8 | 45766 | 0,32 | 72,00 |
| Norway | 12,7 | 63094 | 0,23 | 70 |
| Nya Zeeland | 12,6 | 33930 | 0,33 | 68,1 |
| Portugal | 8,7 | 26174 | 0,67 | 64,2 |
| Spain | 9,4 | 31469 | 0,44 | 62,3 |
| Sweden | 12,5 | 26846 | 0,16 | 62,1 |
| Switzerland | 13,9 | 56670 | 0,41 | 70,90 |
| USA | 13,3 | 51249 | 0,66 | 71,00 |
| MEAN VALUE | 11,8 | 41734 | 0,44 | 64,4 |

## Slide 11
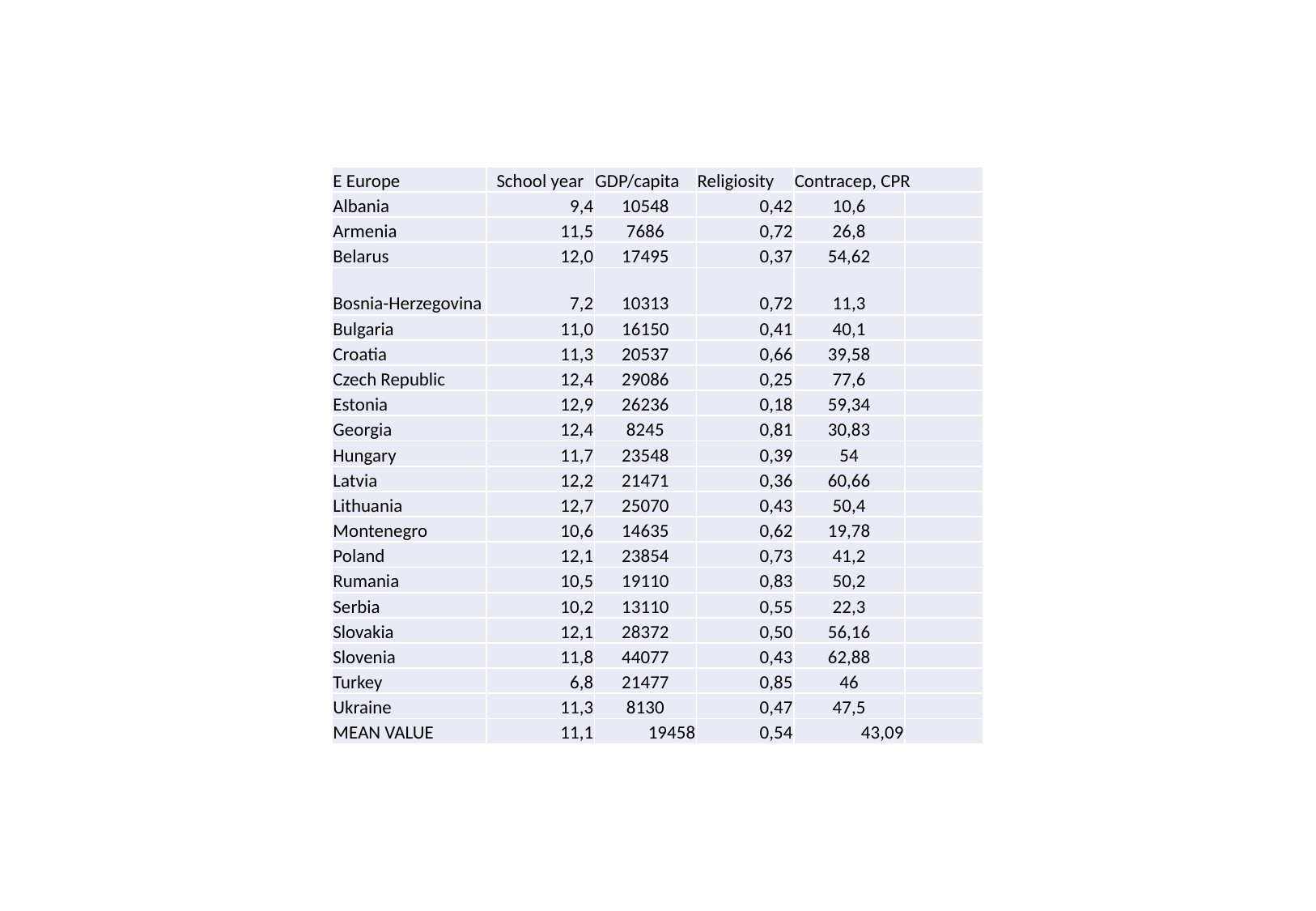

| E Europe | School year | GDP/capita | Religiosity | Contracep, CPR | |
| --- | --- | --- | --- | --- | --- |
| Albania | 9,4 | 10548 | 0,42 | 10,6 | |
| Armenia | 11,5 | 7686 | 0,72 | 26,8 | |
| Belarus | 12,0 | 17495 | 0,37 | 54,62 | |
| Bosnia-Herzegovina | 7,2 | 10313 | 0,72 | 11,3 | |
| Bulgaria | 11,0 | 16150 | 0,41 | 40,1 | |
| Croatia | 11,3 | 20537 | 0,66 | 39,58 | |
| Czech Republic | 12,4 | 29086 | 0,25 | 77,6 | |
| Estonia | 12,9 | 26236 | 0,18 | 59,34 | |
| Georgia | 12,4 | 8245 | 0,81 | 30,83 | |
| Hungary | 11,7 | 23548 | 0,39 | 54 | |
| Latvia | 12,2 | 21471 | 0,36 | 60,66 | |
| Lithuania | 12,7 | 25070 | 0,43 | 50,4 | |
| Montenegro | 10,6 | 14635 | 0,62 | 19,78 | |
| Poland | 12,1 | 23854 | 0,73 | 41,2 | |
| Rumania | 10,5 | 19110 | 0,83 | 50,2 | |
| Serbia | 10,2 | 13110 | 0,55 | 22,3 | |
| Slovakia | 12,1 | 28372 | 0,50 | 56,16 | |
| Slovenia | 11,8 | 44077 | 0,43 | 62,88 | |
| Turkey | 6,8 | 21477 | 0,85 | 46 | |
| Ukraine | 11,3 | 8130 | 0,47 | 47,5 | |
| MEAN VALUE | 11,1 | 19458 | 0,54 | 43,09 | |

## Slide 12
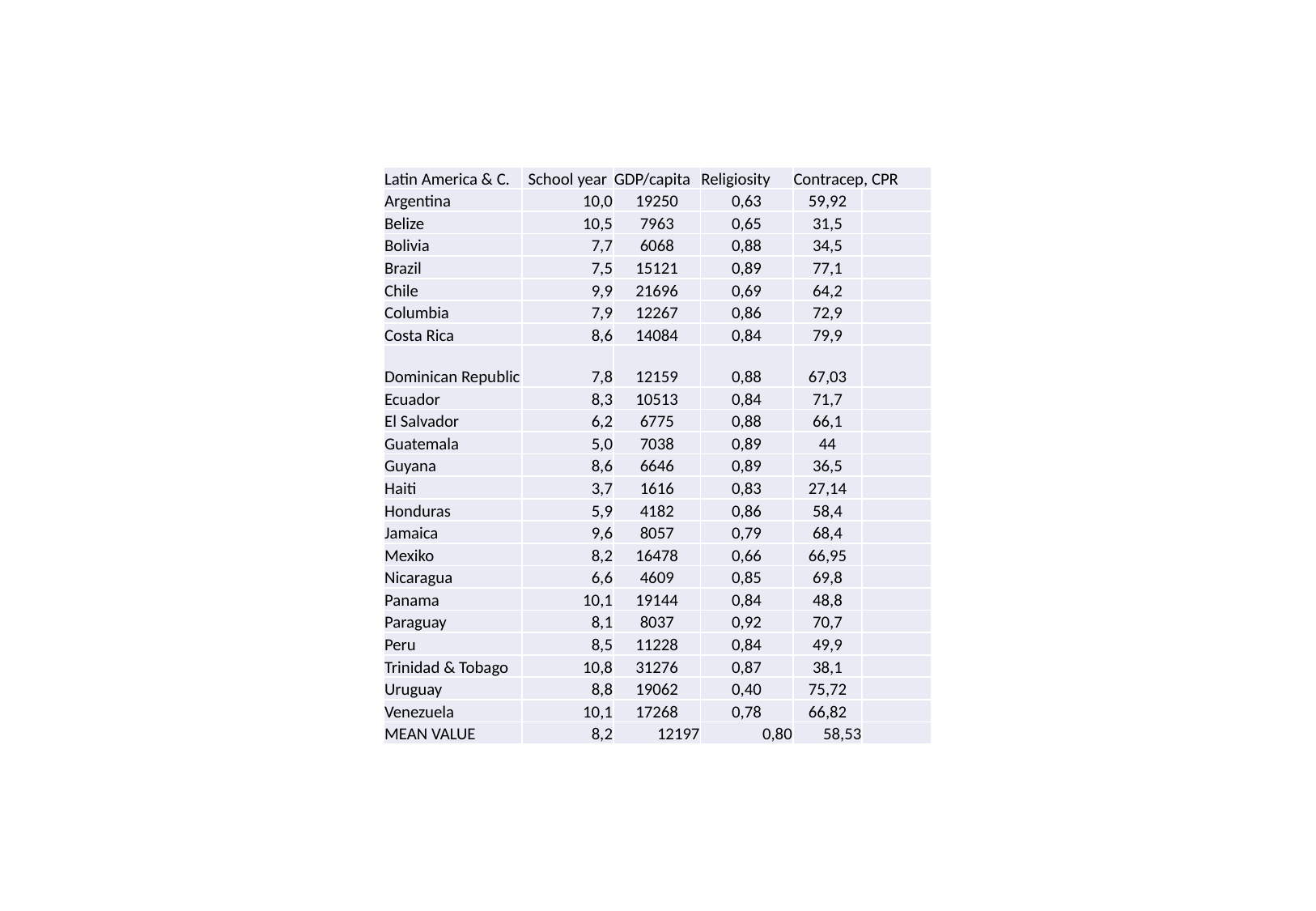

| Latin America & C. | School year | GDP/capita | Religiosity | Contracep, CPR | |
| --- | --- | --- | --- | --- | --- |
| Argentina | 10,0 | 19250 | 0,63 | 59,92 | |
| Belize | 10,5 | 7963 | 0,65 | 31,5 | |
| Bolivia | 7,7 | 6068 | 0,88 | 34,5 | |
| Brazil | 7,5 | 15121 | 0,89 | 77,1 | |
| Chile | 9,9 | 21696 | 0,69 | 64,2 | |
| Columbia | 7,9 | 12267 | 0,86 | 72,9 | |
| Costa Rica | 8,6 | 14084 | 0,84 | 79,9 | |
| Dominican Republic | 7,8 | 12159 | 0,88 | 67,03 | |
| Ecuador | 8,3 | 10513 | 0,84 | 71,7 | |
| El Salvador | 6,2 | 6775 | 0,88 | 66,1 | |
| Guatemala | 5,0 | 7038 | 0,89 | 44 | |
| Guyana | 8,6 | 6646 | 0,89 | 36,5 | |
| Haiti | 3,7 | 1616 | 0,83 | 27,14 | |
| Honduras | 5,9 | 4182 | 0,86 | 58,4 | |
| Jamaica | 9,6 | 8057 | 0,79 | 68,4 | |
| Mexiko | 8,2 | 16478 | 0,66 | 66,95 | |
| Nicaragua | 6,6 | 4609 | 0,85 | 69,8 | |
| Panama | 10,1 | 19144 | 0,84 | 48,8 | |
| Paraguay | 8,1 | 8037 | 0,92 | 70,7 | |
| Peru | 8,5 | 11228 | 0,84 | 49,9 | |
| Trinidad & Tobago | 10,8 | 31276 | 0,87 | 38,1 | |
| Uruguay | 8,8 | 19062 | 0,40 | 75,72 | |
| Venezuela | 10,1 | 17268 | 0,78 | 66,82 | |
| MEAN VALUE | 8,2 | 12197 | 0,80 | 58,53 | |

## Slide 13
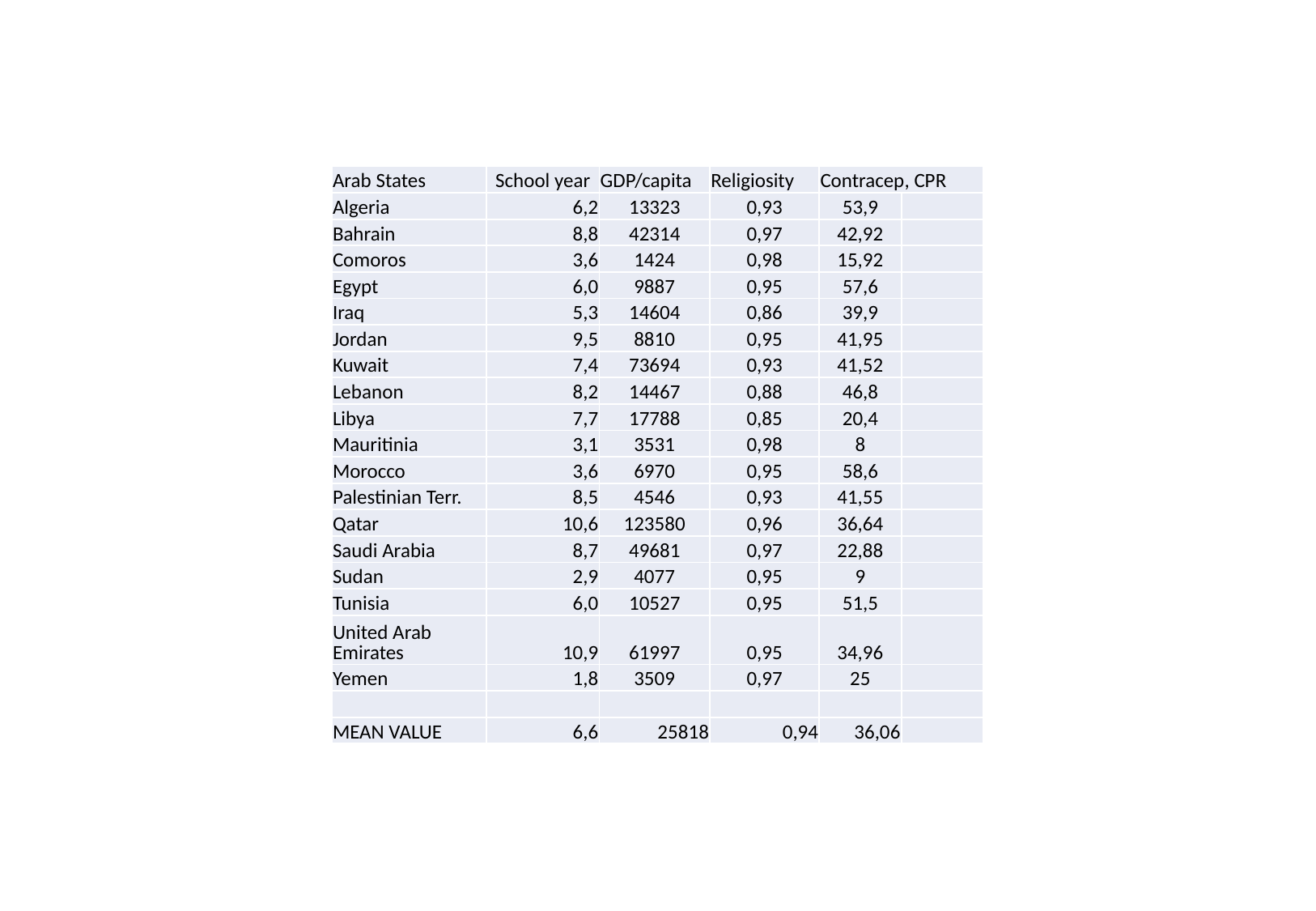

| Arab States | School year | GDP/capita | Religiosity | Contracep, CPR | |
| --- | --- | --- | --- | --- | --- |
| Algeria | 6,2 | 13323 | 0,93 | 53,9 | |
| Bahrain | 8,8 | 42314 | 0,97 | 42,92 | |
| Comoros | 3,6 | 1424 | 0,98 | 15,92 | |
| Egypt | 6,0 | 9887 | 0,95 | 57,6 | |
| Iraq | 5,3 | 14604 | 0,86 | 39,9 | |
| Jordan | 9,5 | 8810 | 0,95 | 41,95 | |
| Kuwait | 7,4 | 73694 | 0,93 | 41,52 | |
| Lebanon | 8,2 | 14467 | 0,88 | 46,8 | |
| Libya | 7,7 | 17788 | 0,85 | 20,4 | |
| Mauritinia | 3,1 | 3531 | 0,98 | 8 | |
| Morocco | 3,6 | 6970 | 0,95 | 58,6 | |
| Palestinian Terr. | 8,5 | 4546 | 0,93 | 41,55 | |
| Qatar | 10,6 | 123580 | 0,96 | 36,64 | |
| Saudi Arabia | 8,7 | 49681 | 0,97 | 22,88 | |
| Sudan | 2,9 | 4077 | 0,95 | 9 | |
| Tunisia | 6,0 | 10527 | 0,95 | 51,5 | |
| United Arab Emirates | 10,9 | 61997 | 0,95 | 34,96 | |
| Yemen | 1,8 | 3509 | 0,97 | 25 | |
| | | | | | |
| MEAN VALUE | 6,6 | 25818 | 0,94 | 36,06 | |

## Slide 14
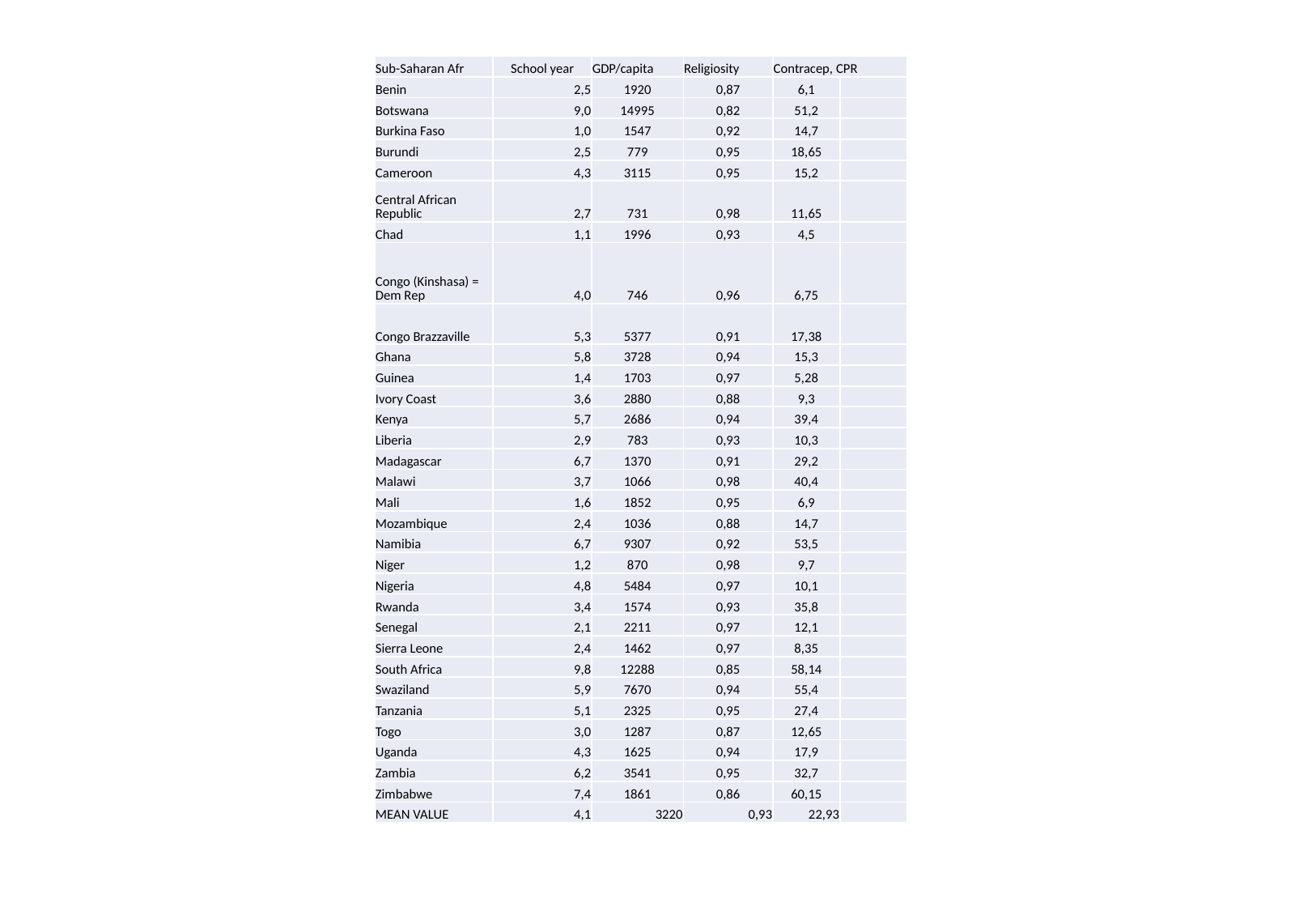

| Sub-Saharan Afr | School year | GDP/capita | Religiosity | Contracep, CPR | |
| --- | --- | --- | --- | --- | --- |
| Benin | 2,5 | 1920 | 0,87 | 6,1 | |
| Botswana | 9,0 | 14995 | 0,82 | 51,2 | |
| Burkina Faso | 1,0 | 1547 | 0,92 | 14,7 | |
| Burundi | 2,5 | 779 | 0,95 | 18,65 | |
| Cameroon | 4,3 | 3115 | 0,95 | 15,2 | |
| Central African Republic | 2,7 | 731 | 0,98 | 11,65 | |
| Chad | 1,1 | 1996 | 0,93 | 4,5 | |
| Congo (Kinshasa) = Dem Rep | 4,0 | 746 | 0,96 | 6,75 | |
| Congo Brazzaville | 5,3 | 5377 | 0,91 | 17,38 | |
| Ghana | 5,8 | 3728 | 0,94 | 15,3 | |
| Guinea | 1,4 | 1703 | 0,97 | 5,28 | |
| Ivory Coast | 3,6 | 2880 | 0,88 | 9,3 | |
| Kenya | 5,7 | 2686 | 0,94 | 39,4 | |
| Liberia | 2,9 | 783 | 0,93 | 10,3 | |
| Madagascar | 6,7 | 1370 | 0,91 | 29,2 | |
| Malawi | 3,7 | 1066 | 0,98 | 40,4 | |
| Mali | 1,6 | 1852 | 0,95 | 6,9 | |
| Mozambique | 2,4 | 1036 | 0,88 | 14,7 | |
| Namibia | 6,7 | 9307 | 0,92 | 53,5 | |
| Niger | 1,2 | 870 | 0,98 | 9,7 | |
| Nigeria | 4,8 | 5484 | 0,97 | 10,1 | |
| Rwanda | 3,4 | 1574 | 0,93 | 35,8 | |
| Senegal | 2,1 | 2211 | 0,97 | 12,1 | |
| Sierra Leone | 2,4 | 1462 | 0,97 | 8,35 | |
| South Africa | 9,8 | 12288 | 0,85 | 58,14 | |
| Swaziland | 5,9 | 7670 | 0,94 | 55,4 | |
| Tanzania | 5,1 | 2325 | 0,95 | 27,4 | |
| Togo | 3,0 | 1287 | 0,87 | 12,65 | |
| Uganda | 4,3 | 1625 | 0,94 | 17,9 | |
| Zambia | 6,2 | 3541 | 0,95 | 32,7 | |
| Zimbabwe | 7,4 | 1861 | 0,86 | 60,15 | |
| MEAN VALUE | 4,1 | 3220 | 0,93 | 22,93 | |

## Slide 15
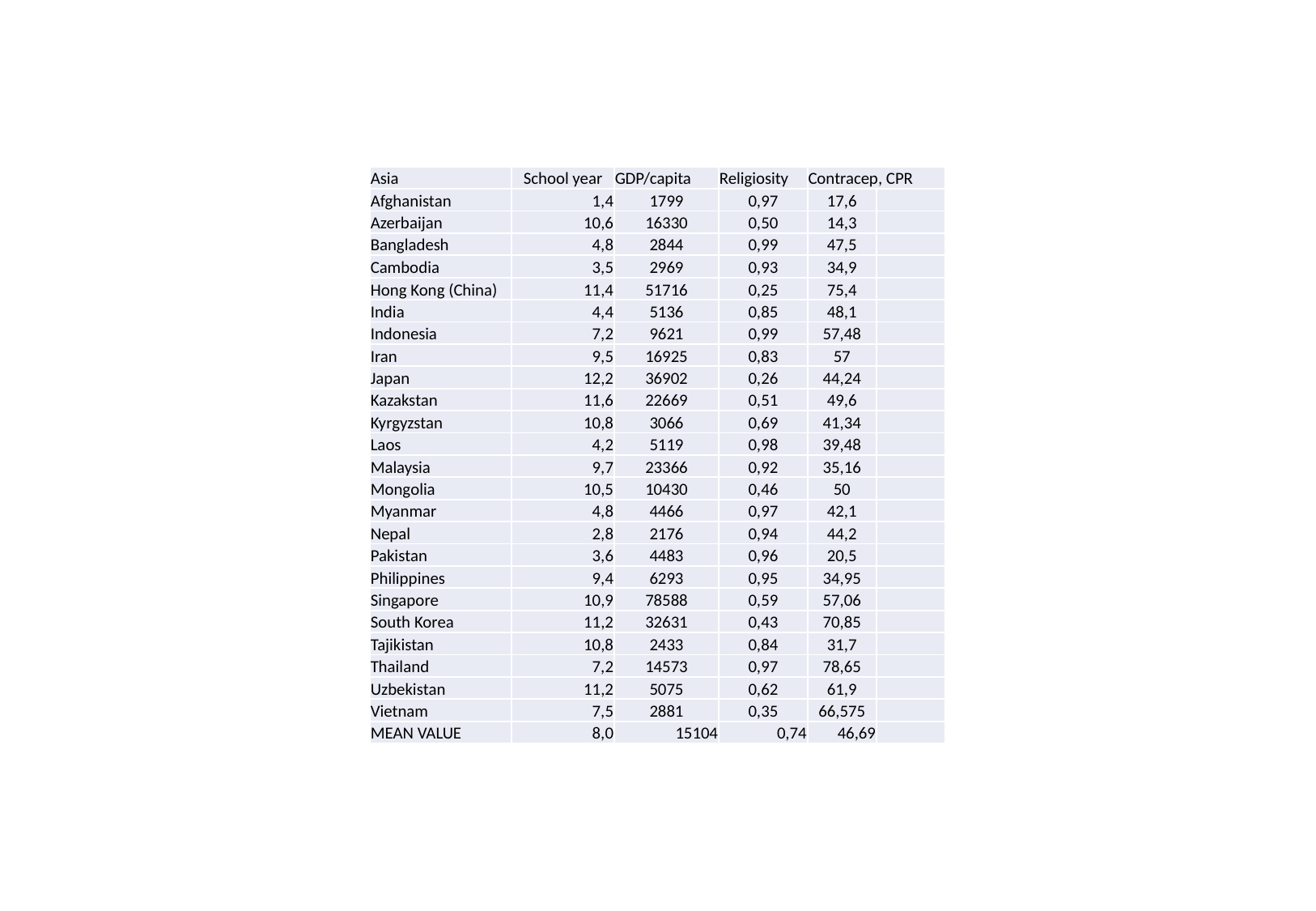

| Asia | School year | GDP/capita | Religiosity | Contracep, CPR | |
| --- | --- | --- | --- | --- | --- |
| Afghanistan | 1,4 | 1799 | 0,97 | 17,6 | |
| Azerbaijan | 10,6 | 16330 | 0,50 | 14,3 | |
| Bangladesh | 4,8 | 2844 | 0,99 | 47,5 | |
| Cambodia | 3,5 | 2969 | 0,93 | 34,9 | |
| Hong Kong (China) | 11,4 | 51716 | 0,25 | 75,4 | |
| India | 4,4 | 5136 | 0,85 | 48,1 | |
| Indonesia | 7,2 | 9621 | 0,99 | 57,48 | |
| Iran | 9,5 | 16925 | 0,83 | 57 | |
| Japan | 12,2 | 36902 | 0,26 | 44,24 | |
| Kazakstan | 11,6 | 22669 | 0,51 | 49,6 | |
| Kyrgyzstan | 10,8 | 3066 | 0,69 | 41,34 | |
| Laos | 4,2 | 5119 | 0,98 | 39,48 | |
| Malaysia | 9,7 | 23366 | 0,92 | 35,16 | |
| Mongolia | 10,5 | 10430 | 0,46 | 50 | |
| Myanmar | 4,8 | 4466 | 0,97 | 42,1 | |
| Nepal | 2,8 | 2176 | 0,94 | 44,2 | |
| Pakistan | 3,6 | 4483 | 0,96 | 20,5 | |
| Philippines | 9,4 | 6293 | 0,95 | 34,95 | |
| Singapore | 10,9 | 78588 | 0,59 | 57,06 | |
| South Korea | 11,2 | 32631 | 0,43 | 70,85 | |
| Tajikistan | 10,8 | 2433 | 0,84 | 31,7 | |
| Thailand | 7,2 | 14573 | 0,97 | 78,65 | |
| Uzbekistan | 11,2 | 5075 | 0,62 | 61,9 | |
| Vietnam | 7,5 | 2881 | 0,35 | 66,575 | |
| MEAN VALUE | 8,0 | 15104 | 0,74 | 46,69 | |

## Slide 16
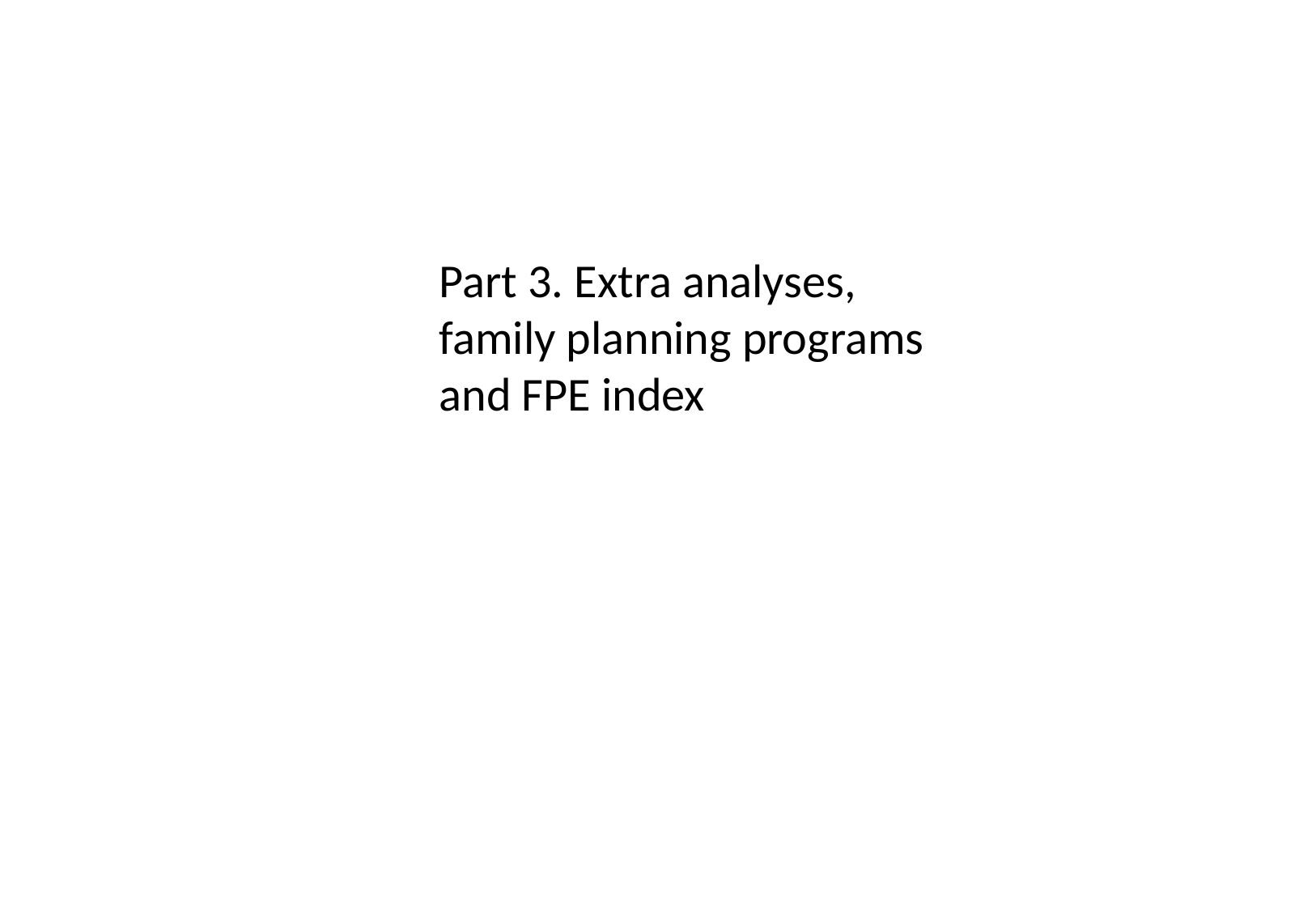

Part 3. Extra analyses,
family planning programs
and FPE index

## Slide 17
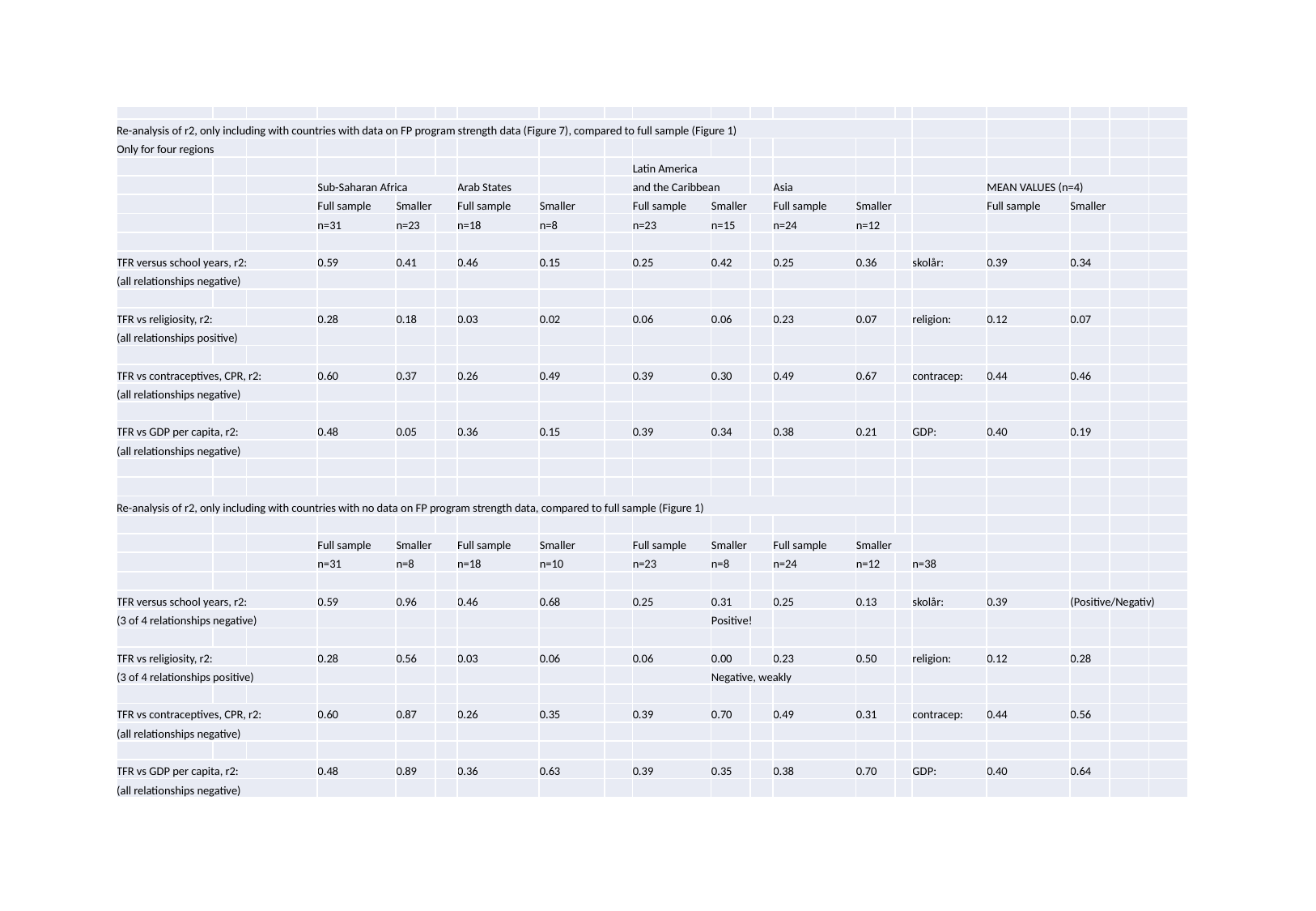

| | | | | | | | | | | | | | | | | | | | |
| --- | --- | --- | --- | --- | --- | --- | --- | --- | --- | --- | --- | --- | --- | --- | --- | --- | --- | --- | --- |
| Re-analysis of r2, only including with countries with data on FP program strength data (Figure 7), compared to full sample (Figure 1) | | | | | | | | | | | | | | | | | | | |
| Only for four regions | | | | | | | | | | | | | | | | | | | |
| | | | | | | | | | Latin America | | | | | | | | | | |
| | | | Sub-Saharan Africa | | | Arab States | | | and the Caribbean | | | Asia | | | | MEAN VALUES (n=4) | | | |
| | | | Full sample | Smaller | | Full sample | Smaller | | Full sample | Smaller | | Full sample | Smaller | | | Full sample | Smaller | | |
| | | | n=31 | n=23 | | n=18 | n=8 | | n=23 | n=15 | | n=24 | n=12 | | | | | | |
| | | | | | | | | | | | | | | | | | | | |
| TFR versus school years, r2: | | | 0.59 | 0.41 | | 0.46 | 0.15 | | 0.25 | 0.42 | | 0.25 | 0.36 | | skolår: | 0.39 | 0.34 | | |
| (all relationships negative) | | | | | | | | | | | | | | | | | | | |
| | | | | | | | | | | | | | | | | | | | |
| TFR vs religiosity, r2: | | | 0.28 | 0.18 | | 0.03 | 0.02 | | 0.06 | 0.06 | | 0.23 | 0.07 | | religion: | 0.12 | 0.07 | | |
| (all relationships positive) | | | | | | | | | | | | | | | | | | | |
| | | | | | | | | | | | | | | | | | | | |
| TFR vs contraceptives, CPR, r2: | | | 0.60 | 0.37 | | 0.26 | 0.49 | | 0.39 | 0.30 | | 0.49 | 0.67 | | contracep: | 0.44 | 0.46 | | |
| (all relationships negative) | | | | | | | | | | | | | | | | | | | |
| | | | | | | | | | | | | | | | | | | | |
| TFR vs GDP per capita, r2: | | | 0.48 | 0.05 | | 0.36 | 0.15 | | 0.39 | 0.34 | | 0.38 | 0.21 | | GDP: | 0.40 | 0.19 | | |
| (all relationships negative) | | | | | | | | | | | | | | | | | | | |
| | | | | | | | | | | | | | | | | | | | |
| | | | | | | | | | | | | | | | | | | | |
| Re-analysis of r2, only including with countries with no data on FP program strength data, compared to full sample (Figure 1) | | | | | | | | | | | | | | | | | | | |
| | | | | | | | | | | | | | | | | | | | |
| | | | Full sample | Smaller | | Full sample | Smaller | | Full sample | Smaller | | Full sample | Smaller | | | | | | |
| | | | n=31 | n=8 | | n=18 | n=10 | | n=23 | n=8 | | n=24 | n=12 | | n=38 | | | | |
| | | | | | | | | | | | | | | | | | | | |
| TFR versus school years, r2: | | | 0.59 | 0.96 | | 0.46 | 0.68 | | 0.25 | 0.31 | | 0.25 | 0.13 | | skolår: | 0.39 | (Positive/Negativ) | | |
| (3 of 4 relationships negative) | | | | | | | | | | Positive! | | | | | | | | | |
| | | | | | | | | | | | | | | | | | | | |
| TFR vs religiosity, r2: | | | 0.28 | 0.56 | | 0.03 | 0.06 | | 0.06 | 0.00 | | 0.23 | 0.50 | | religion: | 0.12 | 0.28 | | |
| (3 of 4 relationships positive) | | | | | | | | | | Negative, weakly | | | | | | | | | |
| | | | | | | | | | | | | | | | | | | | |
| TFR vs contraceptives, CPR, r2: | | | 0.60 | 0.87 | | 0.26 | 0.35 | | 0.39 | 0.70 | | 0.49 | 0.31 | | contracep: | 0.44 | 0.56 | | |
| (all relationships negative) | | | | | | | | | | | | | | | | | | | |
| | | | | | | | | | | | | | | | | | | | |
| TFR vs GDP per capita, r2: | | | 0.48 | 0.89 | | 0.36 | 0.63 | | 0.39 | 0.35 | | 0.38 | 0.70 | | GDP: | 0.40 | 0.64 | | |
| (all relationships negative) | | | | | | | | | | | | | | | | | | | |

## Slide 18
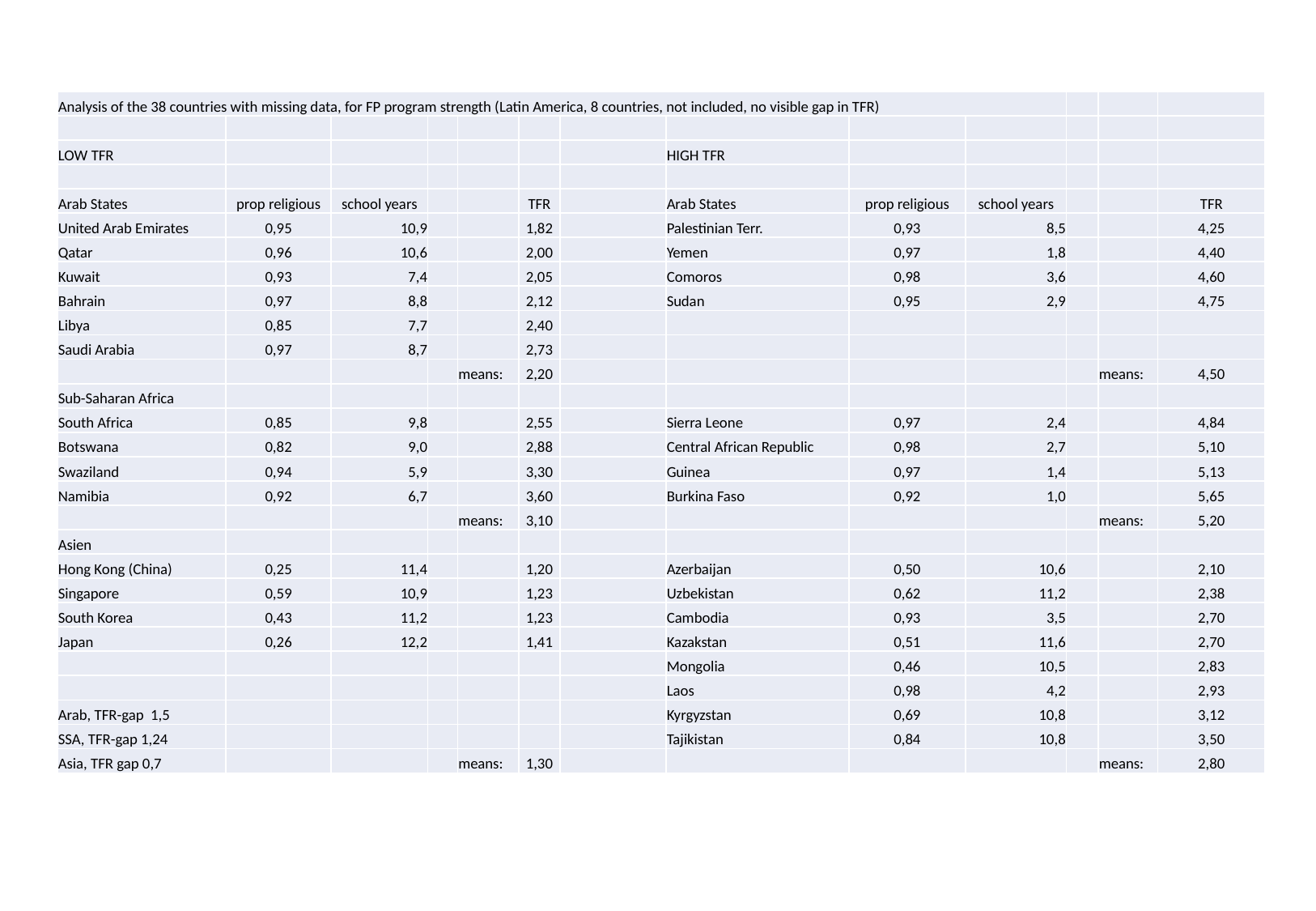

| Analysis of the 38 countries with missing data, for FP program strength (Latin America, 8 countries, not included, no visible gap in TFR) | | | | | | | | | | | | |
| --- | --- | --- | --- | --- | --- | --- | --- | --- | --- | --- | --- | --- |
| | | | | | | | | | | | | |
| LOW TFR | | | | | | | HIGH TFR | | | | | |
| | | | | | | | | | | | | |
| Arab States | prop religious | school years | | | TFR | | Arab States | prop religious | school years | | | TFR |
| United Arab Emirates | 0,95 | 10,9 | | | 1,82 | | Palestinian Terr. | 0,93 | 8,5 | | | 4,25 |
| Qatar | 0,96 | 10,6 | | | 2,00 | | Yemen | 0,97 | 1,8 | | | 4,40 |
| Kuwait | 0,93 | 7,4 | | | 2,05 | | Comoros | 0,98 | 3,6 | | | 4,60 |
| Bahrain | 0,97 | 8,8 | | | 2,12 | | Sudan | 0,95 | 2,9 | | | 4,75 |
| Libya | 0,85 | 7,7 | | | 2,40 | | | | | | | |
| Saudi Arabia | 0,97 | 8,7 | | | 2,73 | | | | | | | |
| | | | | means: | 2,20 | | | | | | means: | 4,50 |
| Sub-Saharan Africa | | | | | | | | | | | | |
| South Africa | 0,85 | 9,8 | | | 2,55 | | Sierra Leone | 0,97 | 2,4 | | | 4,84 |
| Botswana | 0,82 | 9,0 | | | 2,88 | | Central African Republic | 0,98 | 2,7 | | | 5,10 |
| Swaziland | 0,94 | 5,9 | | | 3,30 | | Guinea | 0,97 | 1,4 | | | 5,13 |
| Namibia | 0,92 | 6,7 | | | 3,60 | | Burkina Faso | 0,92 | 1,0 | | | 5,65 |
| | | | | means: | 3,10 | | | | | | means: | 5,20 |
| Asien | | | | | | | | | | | | |
| Hong Kong (China) | 0,25 | 11,4 | | | 1,20 | | Azerbaijan | 0,50 | 10,6 | | | 2,10 |
| Singapore | 0,59 | 10,9 | | | 1,23 | | Uzbekistan | 0,62 | 11,2 | | | 2,38 |
| South Korea | 0,43 | 11,2 | | | 1,23 | | Cambodia | 0,93 | 3,5 | | | 2,70 |
| Japan | 0,26 | 12,2 | | | 1,41 | | Kazakstan | 0,51 | 11,6 | | | 2,70 |
| | | | | | | | Mongolia | 0,46 | 10,5 | | | 2,83 |
| | | | | | | | Laos | 0,98 | 4,2 | | | 2,93 |
| Arab, TFR-gap 1,5 | | | | | | | Kyrgyzstan | 0,69 | 10,8 | | | 3,12 |
| SSA, TFR-gap 1,24 | | | | | | | Tajikistan | 0,84 | 10,8 | | | 3,50 |
| Asia, TFR gap 0,7 | | | | means: | 1,30 | | | | | | means: | 2,80 |
